# Polar reactions of acyclic conjugated bisallenes

**DOI:** 10.3762/bjoc.9.5

**Published:** 2013-01-08

**Authors:** Reiner Stamm, Henning Hopf

**Affiliations:** 1 Institut für Organische Chemie, Technische Universität Braunschweig, Hagenring 30, D-38106 Braunschweig (Germany), Fax: +49 (0)531 / 391 5388

**Keywords:** β-lactams, conjugated bisallenes, cyclopentenones, epoxidation, halogen addition, hydrohalogenation, ionic additions, metalation

## Abstract

The chemical behaviour of various alkyl-substituted, acyclic conjugated bisallenes in reactions involving polar intermediates and/or transition states has been investigated on a broad scale for the first time. The reactions studied include lithiation, reaction of the thus formed organolithium salts with various electrophiles (among others, allyl bromide, DMF and acetone), oxidation to cyclopentenones and epoxides, hydrohalogenation (HCl, HBr addition), halogenation (Br_2_ and I_2_ addition), and [2 + 2] cycloaddition with chlorosulfonyl isocyanate. The resulting adducts were fully characterized by spectroscopic and analytical methods; they constitute interesting substrates for further organic transformations.

## Introduction

Whereas the use of hexa-1,2,4,5-tetraene (**1**) and its derivatives in pericyclic reactions is well documented [2–6], relatively little is known about the behavior of noncyclic, conjugated bisallenes in ionic or polar reactions, whether these involve metalation processes, the addition of halogens and hydrogen halides, or oxidation reactions, to name but a few. To fill this gap we initiated a research program, hoping also that polar reactions of the bisallenes would lead to functionalized derivatives that could be useful for other preparative investigations, just as in the pericyclic case [2–6]. The results of these initial studies are presented here.

**Scheme 1:**
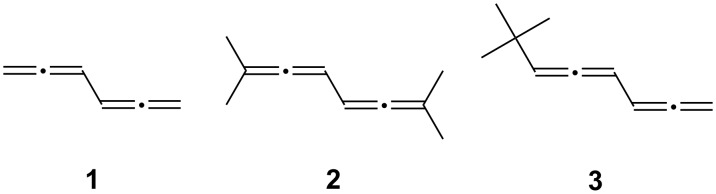
The alkylated conjugated bisallenes **1– 3** as model systems for polar reactions.

Since the parent hydrocarbon **1** (biallenyl) is rather unstable [7], we decided to use the tetramethyl derivative **2** (2,7-dimethylocta-2,3,5,6-tetraene) as a symmetric substrate and the *tert*-butyl derivative **3** (7,7-dimethylocta-1,2,4,5-tetraene) as an asymmetric starting material for our polar reactions (Scheme 1).

Hydrocarbon **2**, prepared by Skattebøl in the early 1960s [3,8–9], is a crystalline solid at room temperature and stable for very long times. Bisallene **3**, first prepared by Ruitenberg, Kleijn, Westmijze, Meijer and Vermeer [10] is decidedly more stable than the parent hydrocarbon **1**: in solution the colorless oil can be kept for weeks at room temperature before discoloration sets in, and in neat form it withstands polymerization for about a week in the deep freezer. The protocols for making these two derivatives from readily available starting materials are straightforward and high-yielding.

## Results and Discussion

### Reactions of **2**/**4** with electrophiles

#### Metalation of **2** and quenching of **4** with simple halides

**Scheme 2:**
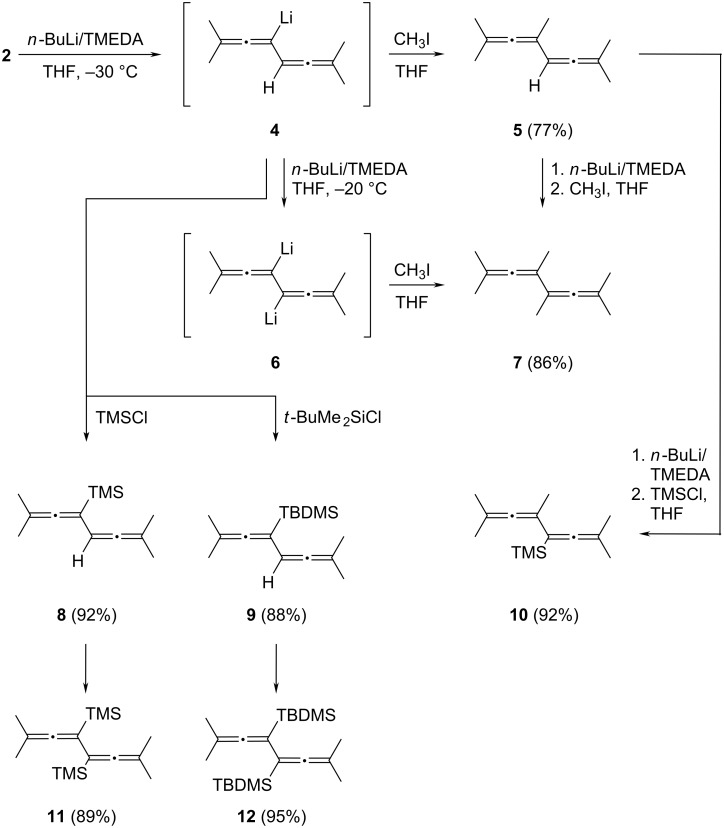
Alkylation and silylation of **2**.

Treating **2** in THF at −30 °C with a slight excess of *n*-butyllithium (1.2 equiv) in the presence of tetramethylethylendiamine (TMEDA) and quenching the formed monoanion **4** with methyl iodide in THF results in the formation of 2,4,7-trimethylocta-2,3,5,6-tetraene (**5**, pentamethylbiallenyl) in good yield (77%) (Scheme 2). The hydrocarbon, an oily substance at room temperature, which solidifies in the deep freezer, is perfectly stable and can be handled easily. It is characterized by its spectroscopic data (see Supporting Information File 1), in particular by a septet at δ 5.58 ppm with ^5^*J* = 2.8 Hz for the remaining allenic proton in the ^1^H NMR spectrum and by two singlets at δ 202.5 and 202.8 ppm for the central allene carbon atoms in the ^13^C NMR spectrum. When the methylation is carried out after 3 equiv of *n*-butyllithium have been added, small amounts of a dimethyl product, i.e., the fully alkylated biallenyl **7** can be isolated, possibly formed via the dilithiated intermediate **6** (up to 15% depending on the exact alkylation conditions). However, since the purification of this product required gas-chromatographic separation, a stepwise approach was adopted to prepare it: the monomethylation product **5** was subjected to the metalation/methylation sequence again and yielded pure **7** in very good yield (86%). This hydrocarbon, symmetric and hence relatively high-melting again (mp 72–75 °C), is a known compound [11]; however, in our hands its spectroscopic properties differed slightly from the published ones (see Supporting Information File 1).

Changing to trimethylsilyl chloride and *tert*-butyldimethylsilyl chloride as quenching reagents resulted in the expected formation of **8** and **9**, which are both produced in very good yields and are oils again. Their structures follow from the spectroscopic data listed in Supporting Information File 1. Since **5**, **8**, and **9** still have a “free” allenic hydrogen atom they can be alkylated/silylated again, yielding **10**, **11**, and **12**, all in excellent yield. The highly symmetric derivatives **11** and **12** are again solids, whereas **10** is an oil at room temperature. We have reported on the X-ray analysis of **11** in an earlier study [12].

#### Metalation of **2** and quenching of **4** with allyl bromide (**13**)

**Scheme 3:**
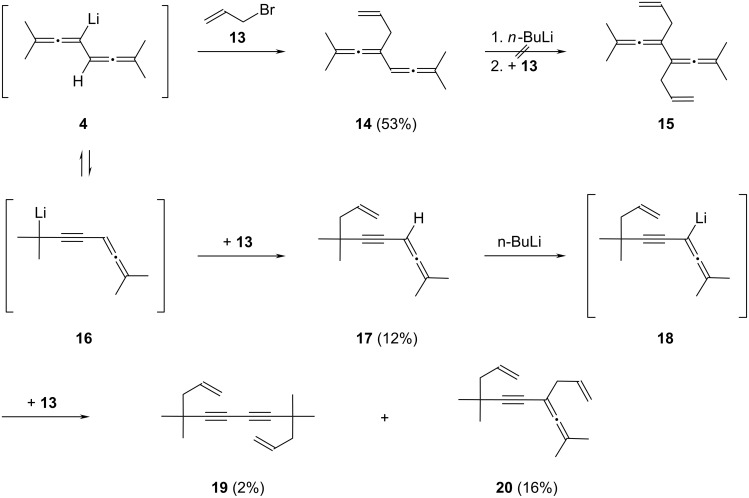
Allylation of the monoanion **4**.

In the next series of experiments **13** was introduced as the quenching reagent for the monolithiated bisallene **4** in the hope of introducing additional unsaturation into the reaction products. Although this goal was accomplished, the overall process is considerably more complex (Scheme 3). Treatment of **2** or **4** in THF at −35 °C with **13** resulted in the formation of four hydrocarbons: two monoallylated products, **14** and **17**, and two bisallylated ones, **19** and **20**, with the relative yields determined by gas chromatography given in Scheme 3; ca. 10% of the reaction mixture was unreacted starting material **2**.

Whereas the structure assignments of **14** and **20** are based on the usual spectroscopic data (see Experimental section; Supporting Information File 1) and are unequivocal, for **17** and **19** only GC–MS data were available, and the given structures are hence speculative and rest only on their mass spectra. Hydrocarbon **14** was obtained in 80% purity by preparative gas chromatography; its complete separation from **17** failed. The bisallyl derivative **20** was isolated in analytically pure form by preparative gas chromatography. All hydrocarbons are colorless oils that, even at −15 °C (deep freeze), are stable for a limited time only.

A simple rationalization of the product formation, in which **14** and **17** are the primary products, is given in Scheme 3. Hydrocarbon **14** is the direct allylation product of **4**, whereas for the generation of **17** we have to assume that its precursor monoanion has the propargyl structure **16**. In all these structures the lithium atom is only meant to mark the position of the carbon atom at which the electrophile attacks; no proposition about the nature of the carbon–lithium bond or the structure of the anion is intended. Further allylation of **14** under the reaction conditions and formation of the symmetric bisallyl derivative of **2**, hydrocarbon **15**, was not observed.

On the other hand, metalation of **17** to the monoanion **18** and its reaction with allyl bromide (**13**) could furnish the bisallyl derivatives **19** and **20**. Note that these reaction pathways are not the only ones conceivable. For example, **14** could also provide **20** if the propargyl anion of the former reacts with **13**.

#### Metalation/trimethylsilylation of **3**

In an attempt to learn about the regioselectivity of the lithiation/quenching process, the asymmetric bisallene **3** was subjected to the above conditions, employing a three-fold excess of the metalation reagent. Compared to the tetramethylderivative **2**, this hydrocarbon possesses five different allenic C–H bonds, which could in principle be replaced by a trimethylsilyl substituent.

**Scheme 4:**
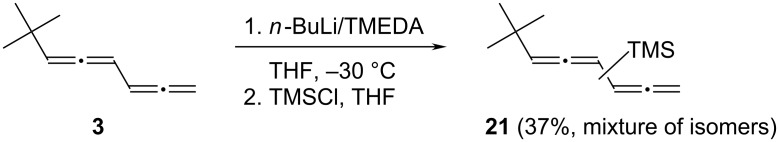
Metalation/silylation of hydrocarbon **3**.

As it turned out, **3** is considerably less reactive than **2**: after comparable reactions times (see Supporting Information File 1) only 37% of the substrate had been converted into products. As shown by GC–MS analysis the reaction mixture contained four mono silylated products (*m*/*z* = 206, [M]^+^) in 1:2.7:3.0:5.3 ratio, whose mass spectra were virtually identical. We therefore assume that the structures of these products are also very similar. Unfortunately, these compounds could not be separated by preparative gas chromatography; even the analytical GC (capillary column) did not show full resolution. Assuming that the hydrogen atom at the substituted end of **3** is not replaced by the TMS-group for steric and electronic reasons, four monosilylated products are possible, i.e., silylation at the unsubstituted end of **3** leading to two diastereomers. General structure **21** (Scheme 4) accounts for our experimental observations.

Regardless of the exact structures of the silylation products **21**, we did not observe any selectivity in this transformation.

#### Quenching of **4** with *N*,*N*-dimethylformamide (DMF) and acetone

Metalated allenes are known to be converted into allenic aldehydes on DMF treatment [13–15].

**Scheme 5:**
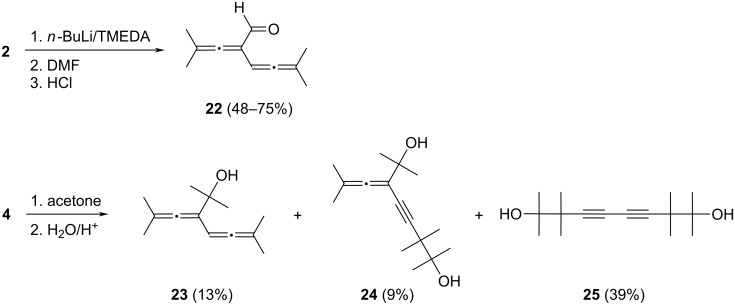
Quenching of **4** with DMF and acetone.

The bisallene **2** reacts analogously: after quenching of the monoanion **4** with DMF and acidic work-up, the bisallenic aldehyde **22** is obtained in 75% yield and 90% purity (GC analysis of the raw product). The compound is apparently not very stable, though: after distillation, **22** is isolated with analytical purity (>99%) as a slightly yellow oil, but with a reduced yield of 48%, the remainder being nonvolatile oligomeric compounds. The structure of the monoaldehyde (see Scheme 5) unambiguously follows from its spectroscopic data, in particular the absorption of the formyl proton at δ 9.49 and the allenic septet at 5.59 ppm with the typical coupling constant ^5^*J* of 2.8 Hz in the ^1^H NMR spectrum. In the ^13^C NMR spectrum the central allenic carbon atoms are revealed by signals at 204.6 and 216.0 ppm; the carbonyl group absorbs at 190.9 ppm.

Replacing the DMF by acetone should lead to a tertiary bisallenic alcohol, and this is indeed the case. However, over all the situation becomes more complex. As illustrated in Scheme 5 three alcohols are produced; their yields are only moderate and become even poorer on separation due to the instability of these products. Again, the conversion is incomplete leading to the recovery of **2** in 25% yield.

The three alcohols produced have the structures **23**–**25** as shown in Scheme 5. According to GC analysis of the raw product mixture they are produced in 13, 9, and 39% yield, respectively. However, only 10, 4, and 8% survive the elaborate and time-consuming separation by distillation, chromatography, and recrystallization.

The analytically pure (>98%) monoalcohol **23**, a colorless oil at room temperature, is the product expected from the reaction of **4** with acetone. The spectroscopic data leave no doubt about its structure. The ^1^H NMR spectrum displays the typical septet (^5^*J* = 3.2 Hz) of the “remaining” allenic hydrogen atom at δ 5.52 ppm. The OH-group absorbs at δ 2.30 ppm, a signal that vanishes after D_2_O-exchange. In the ^13^C NMR spectrum two different central allenic carbon atoms are recognizable again (δ 199.6 and 201.9 ppm), and the saturated tertiary carbon atom absorbs as a singlet at δ 71.5 ppm. All other spectroscopic data (see Supporting Information File 1) also agree with the structure proposal.

The other two alcohols, **24** and **25**, both of them diols, are obviously bisalkylation products and involve the generation of anionic intermediates with a propargyl structure. The “half-rearranged” diol **24** is a slightly yellow oil at room temperature. Structure-defining are the missing allene proton and three signals for carbon atoms not connected to a hydrogen atom: the central allene carbon atom at 210.0 ppm and the two acetylene carbon atoms at δ 72.0 and 75.0 ppm.

The symmetric diol **25** crystallizes from ethanol with one equivalent of included water. Stoichiometric host/guest associates have often been observed for this class of compounds [16]. The NMR spectra of **25** show only a small number of signals due to the symmetry of the molecule. In the ^13^C NMR spectrum the acetylenic (δ 74.2 and 83.8 ppm) and tertiary carbon atoms (δ 41.4 and 66.7 ppm) are clearly visible as singlets. All other spectroscopic details agree with the proposed structure (Supporting Information File 1).

#### Miscellaneous reactions of **2** and **4** with electrophiles

**Scheme 6:**
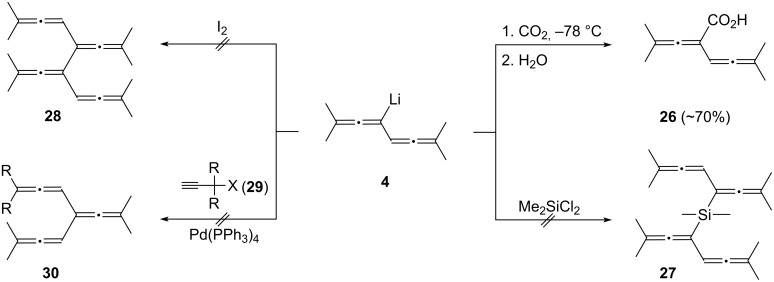
Further reactions of **2**/**4** with various electrophiles.

The carboxylation of simple metalated allenes has often been described in the chemical literature [17]. And the process is apparently successful for **2**/**4** also: the ^1^H NMR spectrum of the raw product, obtained in ca. 70% yield, shows the expected signals at δ 5.59 ppm (^5^*J* = 2.9 Hz) for the remaining allene proton and at 9.5 ppm (broad signal) for the carboxyl group. However, all attempts to isolate the new bisallene derivative **26** resulted in resin formation and destruction of the original signals (Scheme 6).

In another series of experiments we tried to use **4** as a building block for different tri- and tetraallenes. As indicated in Scheme 6 all of these experiments failed. Neither could we prepare the “coupling product” **27** by treatment of **4** with the biselectrophile dimethylysilyl dichloride nor the dimer **28** by the direct action of iodine on the organolithium compound **4**. In this latter case we noted after work-up and GC–MS analysis of the raw product mixture that addition products of **2** had been produced besides mostly polymeric material. We will return to the addition of iodine to **2** later. The coupling of various propargyl halides (**29**, R = H, CH_3_; X = Cl, Br) in the presence of a Pd-catalyst did not lead to the hoped for hydrocarbon **30**.

### Oxidation of conjugated bisallenes

The oxidation of allenes has already been studied previously. In seminal papers Crandall and his students described the epoxidation of differently substituted monoallenes and showed that methylene oxiranes are the initial oxidation products. These, however, are often not isolated but react further either to cyclopropanones (the so-called “allenoxide-cyclopropanone rearrangement”) or are oxidized a second time to provide dioxaspiro[2.2]pentane derivatives [18–20].

As far as functionalized allenes are concerned, the contributions of Bertrand, Grimaldi, and co-workers [21–24] are particular noteworthy in the present context. The French authors demonstrated that vinylallenes, hydrocarbons rather similar to the systems studied here, provide cyclopentenone derivatives in a process mimicking the Nazarov cyclization [25].

**Scheme 7:**
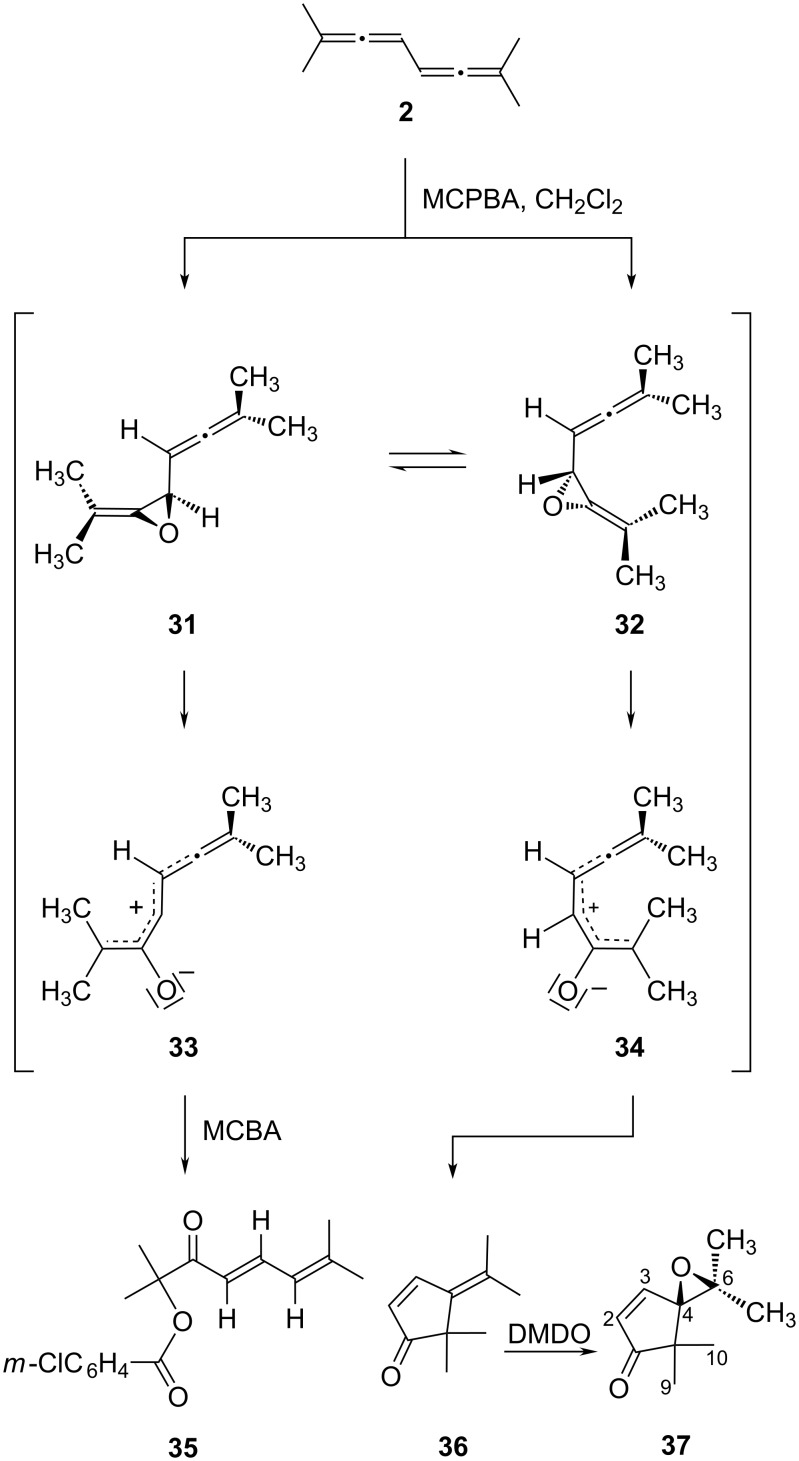
Oxidation of conjugated bisallenes with different oxidizing agents according to [26].

The first conjugated bisallenes, including the tetramethyl derivative **2**, were epoxidized by Pasto et al. [26]. These authors showed that this hydrocarbon provides the products **35** and **36** on treatment with *m*-chloroperbenzoic acid (MCPBA) in dichloromethane (product ratio: 71:29). With excess dimethyldioxirane (DMDO) the epoxide **37** was produced solely, evidently generated by further oxidation of the monooxidation product **36**. The still remaining double bond in **37** is not oxidized under these conditions (Scheme 7).

To rationalize their findings these authors propose monooxidation of **2** with MCPBA to the alleneoxide **31** initially, which is in equilibrium with its conformational isomer **32**. Ring-opening of these strained intermediates then provides the “stretched” and the “closed” zwitterions **33** and **34**, respectively. Whereas the open form **33** is intercepted by the anion derived from the epoxidation reagent, *m*-chlorobenzoic acid (MCBA-anion), to provide **35**, the curved intermediate **34** can easily cycloisomerize to the cyclopentenone **36**. That the nature of the oxidation reagent plays a crucial role in these reactions was demonstrated by oxidation of 8,8-dimethylnona-2,3,5,6-tetraene, which reacts to a complex mixture of cyclopentenone derivatives of type **36** simply on exposure to air (derivative **2** does not react under these conditions) [26].

Since some of the above oxidation products were not fully purified and characterized we decided to investigate the epoxidation of several bisallenes prepared in this study more carefully.

**Scheme 8:**
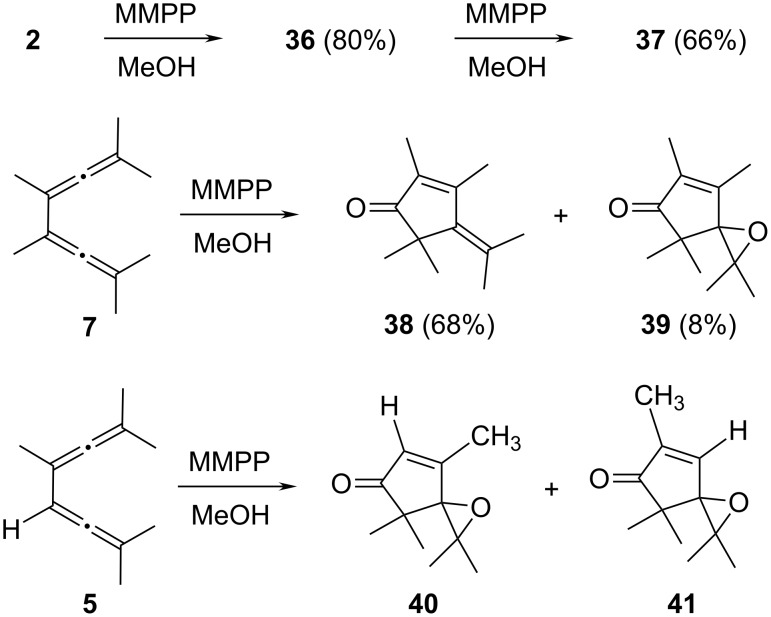
Oxidation of **2, 7** and **5** with MMPP.

The oxidation of the tetramethylbisallene **2** was carried out with magnesium monoperoxy phthalate (MMPP) since we had also observed the formation of addition products of **2** (*m*-chlorobenzoates) when we used MCPBA for epoxidation. Indeed, ketone **36** could be obtained in 80% yield with MMPP (GC analysis), with no other oxidation products being observed (Scheme 8). Compound **36** is a highly volatile ketone, and this may explain the pronouncedly reduced yield (32%) when it was isolated from the product mixture by column chromatography followed by rotary evaporation of the solvent. The spectroscopic data of **36** are identical with those given in the literature [26]. As far as the oxidation mechanism is concerned it agrees with the suggestion of Pasto (see above).

When the epoxidation reagent was employed in excess (fivefold) the epoxide **37** was produced in 66% yield (with reference to **2**) as a colorless oil. Since the analytical and spectroscopic data are incomplete in the literature [26], these data are given in full in Supporting Information File 1.

The next bisallene to be oxidized was the fully substituted hexamethyl derivative **7**, prepared as described in Scheme 2. Reaction with 0.5 equiv of MMPP resulted in the formation of 68% of **38** and a trace amount of the epoxide **39** (8%), while 24% of the substrate was recovered (GC analysis). Since the complete separation of the two products was difficult, the experiment was repeated with excess MMPP (1.5 equiv); in this case the yield of **39** was 62% and the ketoepoxide could be separated and characterized spectroscopically (see Supporting Information File 1). Again, we believe that the Pasto mechanism explains the outcome of the process well.

Lifting the symmetry of the starting material would allow insight into the regioselectivity of the epoxidation process. We therefore took bisallene **5** and subjected it to MMPP treatment (fivefold excess). The reaction took place in acceptable yield (65%) and provided a mixture of two bis oxidation products, **40** and **41**, in 5:1 ratio. Although the two regioisomers were separated on the analytical GC, the attempted preparative GC separation failed because the products did not survive the required column temperature (100 °C). Although the structural difference between the two isomers is rather small, they can be distinguished by the absorption of the remaining olefinic proton in the ^1^H NMR spectrum. In the main isomer **40** this proton (2-H) is right next to the carbonyl group and absorbs at δ 6.18 ppm, whereas in the side product this proton (3-H) appears at δ 6.86 ppm. Both protons couple with the neighboring methyl substituents (q, ^4^*J* = 1.4 and 1.2 Hz, respectively) [27]. Applying the same mechanism as in Scheme 7 leads to the conclusion that it is the more highly substituted allene moiety that is attacked preferentially by the epoxidation reagent.

**Scheme 9:**
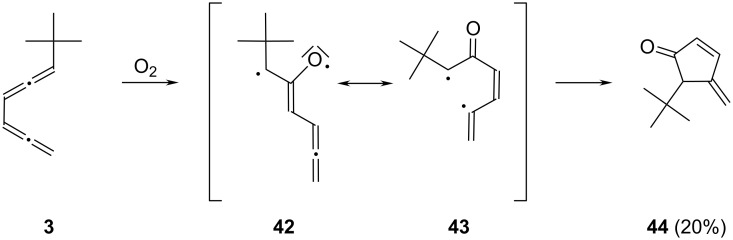
Oxidation of the asymmetric bisallene **3** by air.

Another asymmetric bisallene is the *tert*-butyl derivative **3**. It has already been observed by Pasto and co-workers that *tert*-butylated bisallenes can be oxidized by air (see above) [26]. We therefore kept a solution of **3** in dichloromethane for 4 days under an atmosphere of pure oxygen. The only oxidation product is 5-*tert*-butyl-4-methenyliden-2-cyclopenten-1-one (**44**, Scheme 9), produced in 20% yield. An increase in oxidation time does not result in a yield increase, since the already produced **44**, which is evidently not very stable, starts to form intractable polymers. Typical features of the ^1^H NMR spectrum of this cyclic ketone are the intense singlet at δ 1.00 ppm for the *tert*-butyl substituent and the multiplet of the tertiary ring hydrogen atom at δ 2.50 ppm, which couples with the semicyclic methylene protons. In the ^13^C NMR spectrum this sp^3^-hybridized carbon atom absorbs at δ 57.7 ppm (doublet, low-field shift because of the neighboring carbonyl group, which absorbs at δ 208.6 ppm).

To rationalize our observations we adopt the radical mechanism proposed previously by Pasto [26]. In the first step, oxygen attacks the substrate under formation of the diradical **42**. This, in turn, closes from its resonance structure **43** to yield the isolated product **44**. Had the oxidation taken place at the unsubstituted allene moiety of **3**, the *tert*-butyl substituent would have ended up at the semicyclic double bond according to this mechanism. Since a second *tert*-butyl signal could not be detected in the NMR spectrum of the product mixture, the reaction evidently takes place with very high regioselectivity.

**Scheme 10:**
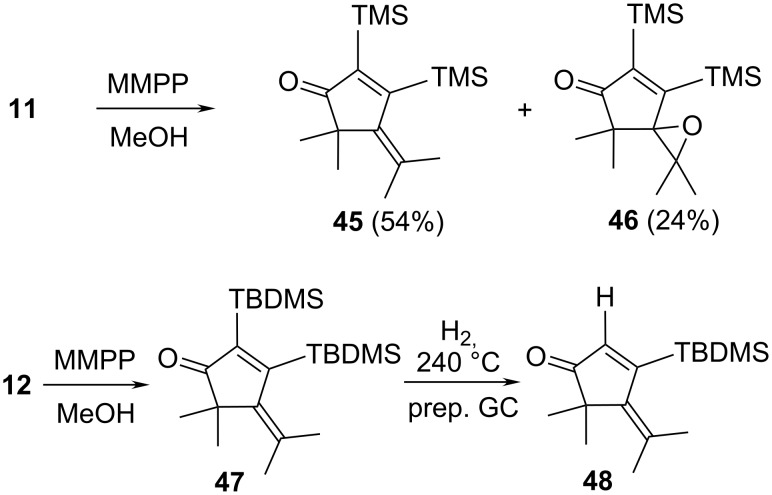
Epoxidation of the disilylbisallenes **11** and **12**.

In a final set of epoxidation experiments the highly hindered silyl derivatives **11** and **12** were oxidized with MMPP (Scheme 10).

Treatment of **11** with MMPP (0.5 equiv) in methanol for 2 days yielded a mixture of the ketone **45** (54%) and the secondary product **46** (24%), with the rest of the product mixture being the unchanged starting material. Since the two products could not be separated by preparative gas chromatography, the oxidation was repeated with 0.7 equiv of MMPP and extended reaction time (10 d, rt). Now all starting material was oxidized and **46** could be isolated by preparative GC in analytically pure form. The spectroscopic data are unremarkable and allow an unambiguous structure assignment (see Supporting Information File 1).

The oxidation of the sterically more shielded **12** was even slower than that of **11**: after 3 days at room temperature only 47% of the substrate had reacted. According to spectroscopic analysis (see Supporting Information File 1) the ketone **47** had been formed in the transformation, i.e., no further oxidation to an epoxide analogous to **46** had taken place. We assume that the double bond in **47** is so highly shielded by the neighboring *tert*-butyldimethylsilyl substituent that it is not attacked by MMPP anymore. To obtain analytically pure material we attempted to purify the raw product by preparative gas chromatography. To realize a sufficiently short retention time, the column temperature had to be increased to 240 °C. Astonishingly the resulting oil, obtained in poor yield (11%) showed a singlet at δ 6.45 ppm, obviously caused by an olefinic proton. This means that during the separation process (which employed hydrogen as the carrier gas) the initial product **47** had lost one of its voluminous substituents. The spectroscopic data (see Supporting Information File 1) suggest that it is **48** that has been produced under the purification conditions.

### Hydrohalogenation and halogenation of acyclic conjugated bisallenes

#### Hydrohalogenation of **2** and **5**

The addition of protic acids HX (with X usually being Cl and Br) to allenes has been studied quite carefully. The process usually takes place according to the Markovnikoff rule and often cannot be stopped at the monoaddition stage [28]. For simple allenes, including the parent hydrocarbon, another side reaction leads to cyclodimerisation products (1,3-dihalo-cyclobutanes) [29]. With hydrochloric and hydrobromic acid, vinylallene (penta-1,2,4-triene) provides (*E*)- and (*Z*)-2-halo-1,3-pentadiene [21,30].

**Scheme 11:**
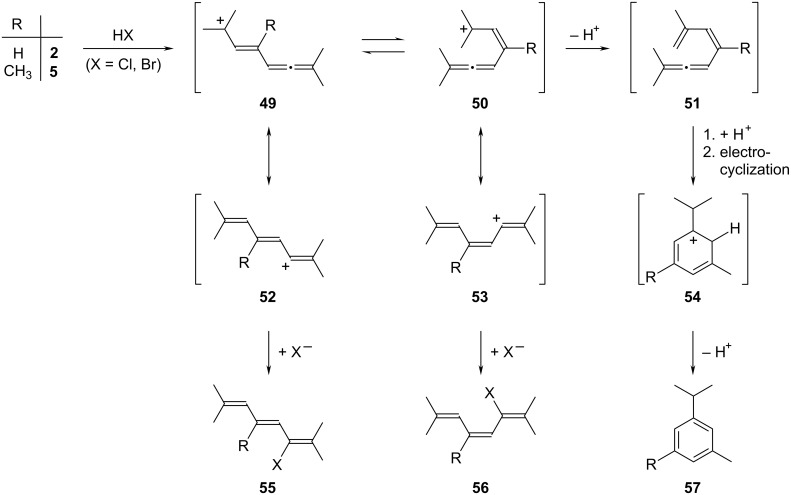
The addition of HCl and HBr to the bisallenes **2** and **5**.

For the reaction of **2** with hydrogen chloride, a solution of the hydrocarbon in diethyl ether was cooled to −70 °C under nitrogen and an excess of HCl gas in ether was added. The temperature was increased to −30 °C, and the solution was then kept at this temperature for 5 h; afterwards it was slowly raised to room temperature. After work-up an oily mixture was obtained, which by preparative gas chromatography could be separated into two components. One component consisted of (*E*)- and (*Z*)-3-chloro-2,7-dimethl-octa-2,4,6-triene (**55** and **56**, together 45%), the other was *m*-cymene (**57**, 20%, Scheme 11).

Whereas **57** could be readily purified by gas chromatography and identified by spectral comparison with the authentic hydrocarbon, the two diastereomers **55** and **56** could not be separated and were analyzed as a mixture. All spectroscopic and analytical data (see Supporting Information File 1) of this mixture point to the structure(s) given in Scheme 11.

To rationalize these findings we propose the following addition mechanism (Scheme 11). The bisallene **2** possesses three different positions to which a proton can be added: the two terminal carbon atoms of the allene moiety and its central carbon atom. Although the two terminal atoms are differently substituted the resulting cations are both of the vinyl type and cannot be stabilized by mesomeric or hyperconjugative effects. This, however, is not the case for the central sp-hybridized carbon atom and the protonation will hence take place there. The resulting cation **49** is tertiary and can be stabilized additionally by interaction with the neighboring (allyl) double bond as well as the remote allene group; a corresponding resonance structure is shown in **52**. Intermediate **49** can adopt two extreme conformations: the stretched (*transoid*) structure **49** and the coiled (*cisoid*) structure **50**. For the latter form we can again formulate a resonance structure, **53**. In principle, both the **49**/**50** and the **52**/**53** pair can equilibrate, but for both pairs we would expect the *transoid* structure to be more stable (less internal steric interaction). When **52** is intercepted by the nucleophile (Cl^−^ in this case) the (*E*)-diastereomer **55** results; likewise **53** furnishes the (*Z*)-isomer **56**. From these considerations we would expect **55** to be the dominant product. If **50** loses a proton the butadienylallene **51** results, which by reprotonation at its allene carbon atom, followed by electrocyclization is converted into **54**. Proton loss of this σ-complex yields the isolated aromatic product **57** in the last step. Thermal cyclizations of tetraenes such as **51** to aromatic compounds have been reported in the literature [31].

For the interception of the cations in Scheme 11 by bromide very similar results are obtained, the ratio of **55** (X = Br) and **56** (X = Br) now being 20:1, and their combined yield amounting to 53% with 27% of *m*-cymene **57** generated as the third, aromatic product. The increase in diastereoselectivity is expected, considering the larger ionic radius of bromide compared to chloride.

The case of the higher, asymmetrically substituted bisallene **5** allowed the investigation of the discrimination between two different allene subunits. Under similar conditions as those above (5 h at −45 °C, then slow temperature increase to room temperature) HCl addition resulted in the formation of four products (GC–MS-analysis of the raw product mixture): two, produced in 9 and 7% yield, were isomers of the starting material. We assume that they are hydrocarbons comparable to **57** (R = CH_3_); however, their separation was unsuccessful. The other two products, produced in 70 and 8% yield, are monochlorides. They could be separated (under considerable material loss) by preparative gas chromatography, and according to their spectral data (see Supporting Information File 1), we assign structures **55** and **56** to them (Scheme 11).

As in the case of the HCl addition to **2**, the UV spectra of the products were particularly valuable for structure assignment; they clearly show that the products obtained possess a conjugated triene chromophore. The formation of the monochlorides very likely proceeds via the carbocationic intermediates **49**/**50** and **52**/**53** (R = CH_3_). For the generation of the aromatic isomers of the substrate we postulate a butadienylallene intermediate, **51**, again.

#### Halogenation of **2**, **3** and **11**

**Scheme 12:**
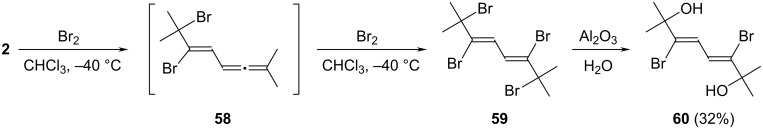
The addition of bromine to the bisallene **2**.

Among the addition of halogens the addition of bromine and iodine to various allenes has been particularly well studied [28]. Whereas bromine is added to the more highly substituted double bond of simple monoallenes, iodine prefers the addition at the less substituted end. Whether the same or a similar mechanistic path is followed in these reactions is unknown. As compounds more related to the bisallenes studied here, several vinyallenes have been treated with one equivalent of bromine to yield adducts in which the most highly substituted double bond has been attacked, leaving the conjugated system unaffected [32–33]. Applied to the conjugated bisallene **2**, this mode of addition should provide either the monoadduct **58** or the bisadduct **59** as the primary product (Scheme 12).

The addition of excess bromine (2 equiv) to **2** was carried out in trichloromethane solution at −40 °C. The GC–MS analysis of the raw product mixture showed that at least three dibromides (monoadducts) and a tetrabromide as well as **57** (R = H) had been formed. However, the actual isolation of any of these products by preparative gas chromatography was unsuccessful. Since two of the bromine substituents in the putative tetrabromide **59** are in allylic positions, we reasoned that we could hydrolyze this adduct to the corresponding diol. We therefore stirred a slurry of **59** in water in the presence of neutral aluminum oxide and indeed could isolate, after work-up, the diol **60** in the form of colorless cubes. The IR spectrum of the compound is dominated by an intense OH absorption band at 3220 cm^−1^; in the ^1^H NMR spectrum both the olefinic (δ 6.99 ppm) and the methyl protons (δ 1.54 ppm) appear as sharp singlets; the UV spectrum reveals the presence of a diene chromophore (see Supporting Information File 1).

Repeating the bromine addition experiment with the fully substituted bisallene derivative **11** also leads to bromine addition as demonstrated by GC–MS analysis of the raw product mixture, but all attempts to separate at least the major adducts by chromatography and/or recrystallization failed.

More is known already about the addition of iodine to conjugated bisallenes. For example, Neumann and Schriewer added iodine to the *meso-*derivative **61** and obtained a diiodide to which they assigned structure **62** [34]. Although the binding sites of the two halogen atoms are apparently correct, the given stereochemistry is disputable, since no unambiguous stereostructure assignment was performed. It is surprising that the *d*,*l*-diastereomer of **61** furnishes the same iodine addition product as the *meso-*compound, as claimed by the authors [33].

Turning to our more symmetric **2**, we prepared the analogous diiodide **63** in very good yield under similar conditions. Note that we were also unable to determine the stereochemistry of the central double bond from our spectroscopic data given in Supporting Information File 1 because of symmetry properties of the product.

**Scheme 13:**
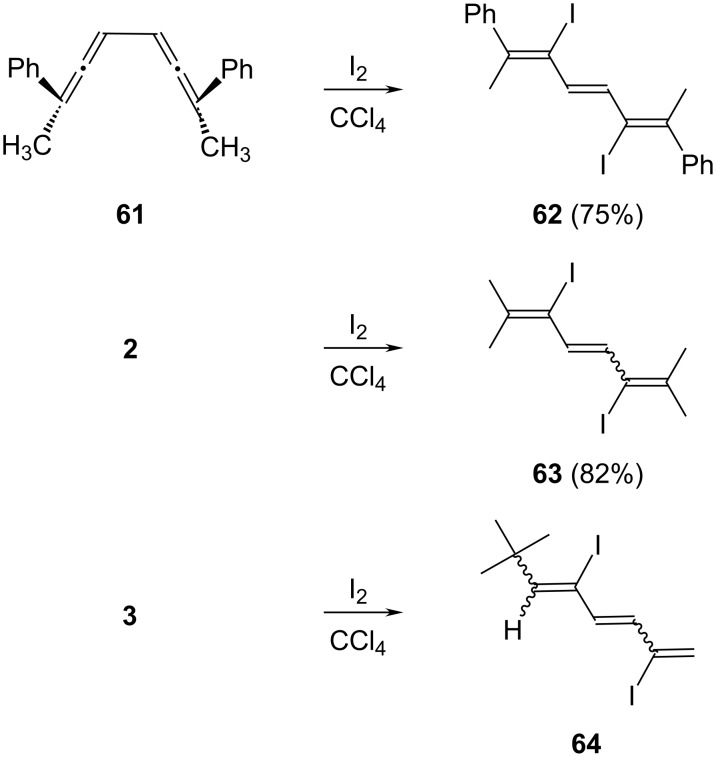
The addition of iodine to the conjugated bisallenes **61**, **2** and **3**.

For the monosubstituted bisallene derivative **3** the situation is stereochemically more complex. Treatment of this chiral compound with iodine in tetrachloromethane at −20 °C caused the formation of two products. To make the formation of bis (or higher) adducts unlikely, we not only treated the hydrocarbon with just one equivalent of the halogen, but always kept its concentration during the addition process very low to reduce the chance of a subsequent halogenation. The two products (symbolized by the general structure **64** in Scheme 13) were obtained with a total yield of 64% and in ca. 15:1 ratio (GC analysis). Although the two products could not be separated, spectroscopic data could be derived for the main component in the product mixture. The protons of the central double bond couple with a coupling constant of 13.5 Hz; this double bond is, hence, very likely *trans*-configured. Since this value is about half way between the (*E*) and (*Z*) coupling constants of other oligoenes [35] an unambiguous decision is difficult on this basis alone. We prefer the (*E*)-configuration, though, since the alternative diastereomer and the path leading to it would be much more sterically hindered. Unfortunately, all other olefinic protons absorbed as (pseudo) singlets (at δ 6.04, 6.44 and 6.59 ppm). We therefore could not assign the orientation between the iodine and the *tert*-butyl group at one of the terminal double bonds.

Also open at the present time is the question of whether the iodine addition takes place by a radical or an ionic mechanism.

#### Addition of chlorosulfonyl isocyanate to **2**

**Scheme 14:**
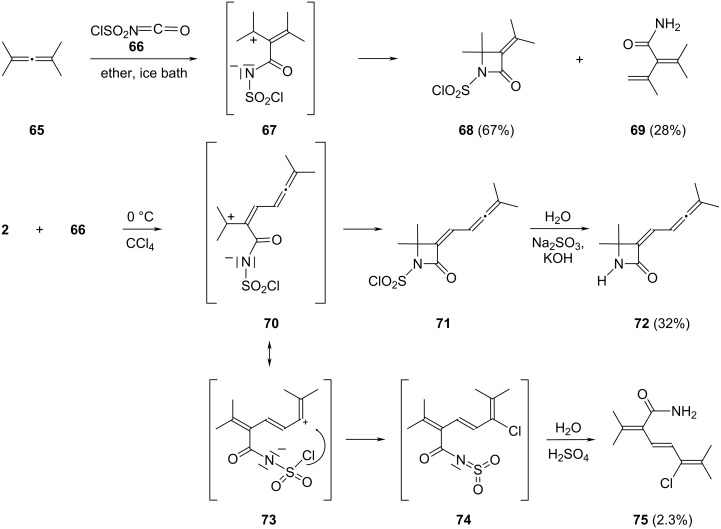
Addition of chlorosulfonyl isocyanate (CSI, **66**) to allenes.

The addition of heterocumulenic systems to several allenes has been studied previously. For instance, Moriconi and Kelly added chlorosulfonyl isocyanate (CSI, **66**) to monoallenes, such as tetramethyallene (**65**, 2,4-dimethylpenta-2,3-diene), at ice-bath temperature and obtained, after aqueous work-up, a mixture of the β-lactam **68** and the cross-conjugated amide **69**; other alkylated allenes reacted similarly (Scheme 14) [36–37].

To rationalize their findings they proposed the initial generation of a zwitterionic intermediate **67**, which subsequently either ring-closes to **68** or is converted into **69** by proton loss and hydrolysis of the chlorosulfonyl group.

When the bisallene **2** was treated with **66** (1 equiv) at 0 °C the cycloaddition was complete after ca. 4 h as shown by monitoring the isocyanate band at 2260 cm^−1^ in the IR spectrum. For work-up the reaction mixture was hydrolyzed with ice water and the organic phase separated. On standing, a small amount of a solid crystallized from the aqueous phase. To this we assign structure **75** according to its spectral data (Supporting Information File 1). Of particular diagnostic importance are its amide bands (3515 and 1679 cm^−1^) in the IR spectrum and the absorption maximum at 284 nm in the electronic spectrum (triene chromophore). The central double bond of the triene system is *trans*-configured (^3^*J* = 15.2 Hz) [35].

The main product is the β-lactam derivate **71**. Since this primary addition product displayed some erratic properties in our hands (sometimes it polymerized, sometimes it survived the work-up), we decided to remove the reactive ClSO_2_ group by hydrolyzing it with sodium sulfite solution under slightly basic conditions (KOH, pH = 8). To the resulting colorless solid we assign structure **72**. The IR spectra of the substrate **71** and the free lactam **72** are similar with the exception of the absorption band for the “free” N–H band at 3430 cm^−1^. The carbonyl group of the lactam is shifted to lower wavenumbers (1728 cm^−1^) as compared to **71** (1760 cm^−1^); all other spectroscopic data (Supporting Information File 1) also agree with the structural proposal **72**.

For the formation of the two reaction products we suggest the pathways given in Scheme 14. The initial adduct of CSI to **2** is again a zwitterion **70**. This either cycloisomerizes to the β-lactam **71** or undergoes an intramolecular chlorine shift from its resonance structure **73** to provide the sulfenimide intermediate **74**. Hydrolysis of the latter then provides product **75**.

In another heterocumulene addition Skattebøl and Boanhave studied the addition of dichloroketene and diphenylketene to **2** [38]. In both cases [2 + 2] cycloaddition to one of the “inner” double bonds of the bisallene took place, yielding a 2-methylenecyclobutanone derivative as the reaction product. No further addition to a 2:1 adduct was observed.

## Conclusion

The behavior of several conjugated bisallenes, notably the symmetric tetramethyl derivative **2** and the asymmetric *tert*-butyl compound **3**, in various polar substitution and addition processes has been studied. The allene protons of these compounds can be readily substituted by alkyl and trisalkylsilyl substituents as well as functional groups (allyl, formyl, carboxyl) by a metalation/electrophilic quenching protocol. The oxidation of these highly unsaturated compounds with magnesium monoperoxyphthalate (MMPP) results predominantly in the formation of cyclopentenone derivatives in a Nazarov-type cyclization. The addition of hydrohalides leads to halo-1,3,5-trienes whereas bromine and iodine addition furnish conjugated dihalodienes and trienes. Finally, the reaction of **2** with chlorosulfonyl isocyanate provides a β-lactam derivative formed in a formal [2 + 2] cycloaddition via a zwitterionic intermediate.

Taken together, these studies show that conjugated bisallenes [39], which are readily available by high-yielding synthetic transformations from simple substrates, are useful starting materials for the preparation of a plethora of novel organic compounds.

## Supporting Information

File 1 Experimental part.
