# Supplementary material for: Polar reactions of acyclic conjugated bisallenes
Source: Beilstein J Org Chem. 2013 Jan 8;9:36–48. doi: 10.3762/bjoc.9.5 (PMC3566852; doi:10.3762/bjoc.9.5)
Supplement: File 1 — Experimental part. [file Beilstein_J_Org_Chem-09-36-s001.pdf]

Supporting Information

for

Polar reactions of acyclic conjugated bisallenes

Reiner Stamm and Henning Hopf*

Address: Institut für Organische Chemie, Technische Universität Braunschweig, Hagenring 30, D-38106 Braunschweig (Germany), Fax: +49 (0)531 / 391 5388

Email: Henning Hopf - H.Hopf@tu-bs.de

*Corresponding author

Experimental part

a) General: *Chromatography*: DSC: Polygram Sil G/UV and Polygram Alox N/UV (Macherey & Nagel); column chromatography: Kieselgel 60 (70–230 mesh, Merck/Darmstadt); analytical GC: DANY 3800, SE-30 cap. column, 30 m; preparative GC: Varian Aerograph 920 and Shimadzu GC-8a; columns: see individual procedures below). - *IR spectroscopy*: Perkin-Elmer 1420 and Nicolet 320 FTIR. *UV spectroscopy*: Beckman UV 5230. *Mass spectrometry*: Finnigan 8430 (70 eV) and Finnigan MAT 4515 (40 eV) coupled with Carlo Erba HRGC 5160. *^{1}H and ^{13}C NMR spectroscopy*: Bruker AM 400 at 400.1 and 100.6 MHz, resp., int. TMS. 2,7-dimethylocta-2,3,5,6-tetraene (**2**) [1,2] and 7,7-dimethyl-octa-1,2,4,5-tetraene (**3**) [3] were prepared as described in the literature.

b) Preparation of bisallenes **5**, **7**, **8**, **9**, **10**, **11**, and **12**:

General procedure 1 (GP-1): To a solution of the respective bisallene (5 mmol) in anhydrous THF (50 mL) was added under nitrogen 0.7 g (6 mmol) of tetramethylethylendiamine (TMEDA). The solution was cooled to −30 °C and a solution of *n*-butyllithium in hexane (6 mmol, 1.2–1.6 mol) was added. The solution turned yellow initially and was dark brown after 3 h. To this mixture was added the respective electrophile (see below) at −35 °C, whereupon the solution decolorized. The reaction mixture was warmed to room temp. overnight, taking aliquots which, after hydrolysis, were analyzed by analytical gas chromatography for the progress of the reaction. For work-up the mixture was hydrolyzed (100 mL ice water), followed by addition of pentane (40 mL). The aqueous phase was separated and washed twice with 20 mL portions of pentane. The combined organic phases were dried ($MgSO_4$) and the solvent was removed by rotary evaporation (room temp.). The ultimate work-up is described below for the different quenching experiments.

2,4,7-Trimethylocta-2,3,5,6-tetraene (**5**): The bisallene **2** (670 mg, 5 mmol) was lithiated according to GP-1 and the formed carbanion quenched with methyl iodide (71 mg, 5 mmol). The raw product was purified by kugelrohr distillation (bp ca. 25 °C, 0.5 mbar): 566 mg

(77%) of **5**, colorless oil. IR (film) ῦ = 2983 (s), 2953 (s), 2910 (s), 2855 (s), 1995 (w), 1965 (w), 1445 (m), 1185 (m), 1009 (m), 819 cm^{-1} (m). UV (acetonitrile) λ_{max} (lg ε) = 214 (4.12), 286 (2.46), 309 nm (2.16). ^1H NMR ($CDCl_3$) δ 1.66 (s, 3H, int. CH_3), 1.69 (s, 6H, ext. CH_3), 1.74 (d, ^5J = 2.8 Hz, 6H, ext. CH_3), 5.58 ppm (sept, ^5J = 2.8 Hz, 1H, allene H). ^{13}C NMR ($CDCl_3$) δ 16.8 (q, 1C), 20.7, 20.8 (q, 4C), 92.8 (d, 1C, allene CH), 94.9, 95.1, 98.4 (s, 3C, allene C), 202.5, 202.8 ppm (s, central allene C). MS (70 eV) *m*/*z* (%): 149 (12), 148 (86) $[M]^+$, 147 (11), 133 (72), 115 (21), 105 (100), 93 (30), 91 (68), 81 (43), 79 (51), 77 (36). $C_{11}H_{16}$ (148.25): calcd C 89.19, H 10.81; found, C 89.04, H 10.75.

2,4,5,7-Tetramethylocta-2,3,5,6-tetraene (**7**): According to GP-1 from **5** (740 mg, 5 mmol) and methyl iodide (71 mg, 5 mmol). After solvent (pentane) removal the remaining solid was recrystallized from pentane: 700 mg (86%) of **7**, colorless needles, mp 72–75 °C. IR (KBr): ῦ = 3441 (s), 3436 (s), 3430 (s), 2981 (m), 1639 (m), 1437 (s), 1362 (s), 1050 (s), 570 cm^{-1} (m). UV (acetonitrile) λ_{max} (lg ε) = 203 (4.19), 223 (4.12), 235 nm (3.91). ^1H NMR ($CDCl_3$) δ 1.68 (s, 6H, int. CH_3), 1.70 (s, 12H, ext. CH_3). ^{13}C NMR ($CDCl_3$) δ 17.9 (q, 2C, int. CH_3), 20.9 (q, 4C, ext. CH_3), 96.4 (s, 2C, terminal allene C), 98.5 (s, 2C, int. allene C), 201.7 ppm (s, 2C, central allene C). MS (70 eV) *m*/*z* (%): 163 (13), 162 (100) $[M]^+$, 147 (46), 119 (45), 107 (18), 91 (27), 81 (59), 79 (29), 53 (34), 41 (46). $C_{12}H_{18}$ (126.27): calcd C 88.89, H 11.11; found, C 88.70, H 11.07. The compound has been described in the literature [4], although with incomplete spectroscopic data.

2,7-Dimethyl-4-trimethylsilylocta-2,3,5,6-tetraene (**8**): According to GP-1 from **2** (670 mg, 5 mmol) and trimethylsilyl chloride (550 mg, 5 mmol). The raw product was purified by kugelrohr distillation (bp 25 °C, 0.1 mbar), colorless liquid, 948 mg (92%). IR (CCl_4): ῦ = 2980 (s), 2960 (s), 1950 (m), 1930 (m), 1450 (s), 1375 (s), 1250 (s), 910 (m), 855 cm^{-1} (s). UV (acetonitrile) λ_{max} (lg ε): 210 (4.31), 230 (4.09), 262 nm (3.18, sh). ^1H NMR ($CDCl_3$) δ 0.06 (s, 9H, TMS), 1.66 (s, 6H, ext. CH_3), 1.70 (d, ^5J = 3.3 Hz, 6H, ext. CH_3), 5.61 ppm (sept,

^5J = 3.1 Hz, 1H, allene proton). ^{13}C NMR ($CDCl_3$) δ −1.2 (q, 3C, TMS), 20.0 (q, 2C, ext. CH_3), 22.5 (q, 2C, ext. CH_3), 90.2 (d, 1C, int. allenic C(H),90.3, 93.8, 96.6 (s, 3C, int. allene C), 204.3 and 207.6 ppm (s, 2C, central allene C). MS (70 eV) *m*/*z* (%): 206 (12) $[M]^+$, 132 (7), 117 (6), 97 (20), 73 (100), 45 (14). HRMS: calcd 206.1491; found, 206.1491.

4-*tert*-Butyldimethylsilyl-2,7-dimethylocta-2,3,5,6-tetraene (**9**): According to GP-1 from **2** (670 mg, 5 mmol) and *tert*-butyldimethylsilyl chloride (758 mg, 5 mmol). The raw product was purified by kugelrohr distillation (bp 25 °C, 0.1 mbar), colorless oil, 1.1 g (88%). IR (film): ῦ = 2981 (m), 2956 (s), 2929 (vs), 1957 (m), 1924 (m), 1470 (m), 1322 (s), 1246 (m), 821 cm^{-1} (s). UV (acetonitrile) λ_{max} (lg ε) : 226 (4.43), 230 (4.42), 274 nm (3.78). ^{1}H NMR ($CDCl_3$) δ 0.00 (s, 6H, $Si(CH_3)_2$), 0.85 (s, 9H, $Si(CH_3)_3$), 1.62 (s, 6H, ext. CH_3), 1.63 (d, ^5J = 3.0 Hz, 6H, ext. CH_3), 5.57 ppm (sept, ^5J = 3.0 Hz, 1H, allene proton). ^{13}C NMR ($CDCl_3$) δ −5.3 (q, 2C, $Si(CH_3)_2$), 17.4 (s, 1C, *C*$(CH_3)_3$), 19.9 (q, 2C, ext. CH_3), 20.8 (q, 2C, ext. allenic CH_3), 26.8 (q, 3C, *t*-Bu), 89.7 (s, 1C, ext. allenic C), 91.0 (d, 1C, int. allenic C(H), 91.3 (s, 1C, int. allenic C), 96.2 (s, 1C, ext. allenic C), 204.5 and 209.3 ppm (s, 2C, central allenic C). MS (70 eV) *m*/*z* (%): 248 (10) $[M]^+$, 192 (11), 191 (22), 86 (64), 84 (100), 73 (40). HRMS: calcd 248.1960; found, 248.1960.

2,5,7-Trimethyl-4-trimethylsilylocta-2,3,5,6-tetraene (**10**): According to GP-1 from **5** (740 mg, 5 mmol) and trimethylsilyl chloride (550 mg, 5 mmol). The raw product was purified by kugelrohr distillation (bp 25 °C, 0.2 mbar), colorless oil, 1.01 g (92%). IR (film): ῦ = 2979 (s), 2956 (m), 2932 (s), 2905 (s), 1959 (w), 1927 (m), 1445 (m), 1333 (m), 1245 (s), 630 cm^{-1} (s). UV (acetonitrile) λ_{max} (lg ε): 208 (4.45), 234 nm (4.22, sh). ^{1}H NMR ($CDCl_3$) δ 0.00 (s, 9H, TMS), 1.62 (s, 12H, ext. CH_3), 1.65 ppm (s, 3H, int. CH_3). ^{13}C NMR ($CDCl_3$) δ −0.3 (q, 3C, TMS), 20.1 (s, 1C, int. CH_3), 20.9 (q, 2C, ext. allenic CH_3), 21.6 (q, 2C, ext. allenic CH_3), 92.4 and 95.3 (s, 2C, ext. allenic C), 96.9 and 98.3 (s, 2C, ext. allenic C), 204.2 and 207.3 ppm (s, 2C, central allenic C). MS (70 eV) *m*/*z* (%): 220 (34) $[M]^+$, 205 (11), 147

(11), 97 (38), 91 (16), 81 (27), 73 (100). HRMS: calcd 220.1647; found, 220. 1647. $C_{14}H_{24}Si$ (220.43): calcd C 76.36, H 10.91, Si 12.73; found, C 76.33, H 10.87, Si 12.71.

2,7-Dimethyl-4,5-di(trimethylsilyl)octa-2,3,5,6-tetraene (**11**): According to GP-1 from **8** (1.03 g, 5 mmol) and trimethylsilyl chloride (550 mg, 5 mmol). The raw product was purified by recrystallization from pentane, colorless needles, mp 72–73 °C, 1.20 g (89%). IR (KBr): ῦ = 2973 (s), 2959 (s), 2931 (s), 2899 (s), 1949 (w), 1244 (s), 862 (vs), 707 cm^{-1} (s). UV (acetonitrile) λ_{max} (lg ε): 208 (4.49), 232 (4.22), 258 nm (3.11). ^{1}H NMR ($CDCl_3$) δ 0.07 (s, 18H, TMS), 1.67 ppm (s, 12H, allenicCH_3). ^{13}C NMR ($CDCl_3$) δ −1.5 (q, 6C, TMS), 19.5 (q, 4C, CH_3), 89.3 (s, 2C, ext. allenic C), 93.4 (s, 2C, int. allenic C), 206.7 ppm (s, 2C, central allenic C). MS (70 eV) *m*/*z* (%): 278 (100) $[M]^+$, 263 (21), 257(16), 205 (20), 175 (21), 105 (21), 97 (36), 83 (20), 77 (52), 73 (46). HRMS: calcd 278.1886; found, 278. 1886. $C_{16}H_{30}Si_2$ (278.59): calcd C 69.06, H 10.79, Si 20.15; found, C 69.15, H 10.69, Si 20.16.

4,5-Di(*tert*-butyldimethylsilyl)-2,7-dimethylocta-2,3,5,6-tetraene (**12**): According to GP-1 from **9** (1.28 g, 5 mmol) and *tert*-butyldimethylsilyl chloride (785 mg, 5 mmol). The raw product was purified by recrystallization from pentane, colorless needles, mp. 99 °C, 1.72 g (95%). IR (KBr): ῦ = 2929 (s), 2897 (s), 2854 (s), 1940 (m), 1246 (s), 830 cm^{-1} (vs). - UV (acetonitrile): λ_{max} (lg ε) = 207 nm (4.45). ^{1}H NMR ($CDCl_3$) δ 0.00 (s, 12H, $Si(CH_3)_2$), 0.85 (s, 18H, *t*-Bu) 1.58 ppm (s, 12H, allenicCH_3). ^{13}C NMR ($CDCl_3$) δ −4.7 (q, 4C, $Si(CH_3)_2$), 17.6 (s, 2C, *C*(CH_3)$_3$), 20.3 (q, 4C, allenic CH_3), 27.1 (q, 6C, *t*-Bu), 89.5 (s, 2C, ext. allenic C), 92.1 (s, 2C, int. allenic C), 208.9 ppm (s, 2C, central allenic C). MS (70 eV) *m*/*z* (%): 362 (100) $[M]^+$, 305 (15), 250 (13), 249 (46), 217 (21), 155 (14), 73 (60). $C_{22}H_{42}Si_2$ (362.75): calcd C 72.86, H 11.67, Si 15.49; found, C 72.83, H 11.60, Si 15.41.

c) Allylation of **2**: Preparation of **14**, **17**, **19**, **20**: To a solution of **2** (1.34 g, 10 mmol) and TMEDA (1.38 g, 12 mmol) in anhydrous THF (40 mL) was added under nitrogen *n*-butyllithium solution in hexane (9.6 mL, 12 mmol, 1.25 M) at −35 °C. After stirring for 2 h at

this temperature, allyl bromide (14.5 g, 12 mmol) was added dropwise. The solution was warmed to room temp. overnight, water was added (10 mL), and the organic phase was washed with two 10 mL portions of water. After phase separation, the organic phase was dried ($MgSO_4$) and the product mixture analyzed by GC–MS. The chromatogram showed 5 peaks to which we assign the following structures: substrate **2** (10%; identified by comparison with authentic material), **17** (12%), **14** (53%), **20** (16%), **19** (2%; increasing retention times). MS (70 eV): **14**: *m*/*z* (%) = 174 (8) $[M]^+$, 159 (80), 144 (20), 131 (43), 129 (42), 117 (35), 115 (33), 105 (56), 91 (100), 79 (25), 77 (53), 67 (17), 65 (33), 41 (74); **17**: *m*/*z* (%) = 174 (90) $[M]^+$, 159 (36), 144 (29), 131 (55), 117 (49), 115 (38), 105 (49), 91 (100), 79 (25), 77 (53), 65 (32), 41 (68); **20**: *m*/*z* (%) = 214 (12) $[M]^+$, 173 (30), 158 (26), 143 (100), 131 (70), 129 (41), 128 (39), 117 (36), 116 (25), 115 (50), 91 (75), 77 (42), 41 (65); **19**: *m*/*z* (%) = 214 (1) $[M]^+$, 174 (88), 159 (33), 144 (28), 131 (55), 129 (40), 117 (50), 115 (35), 105 (48), 91 (100), 79 (25), 77 (52), 41 (68).

Subsequent preparative GC separation (SE 54, 3 m, 120–180 °C) allowed the enrichment of **14** to ca. 80% purity (analysis by cap. GC) and the isolation of **20** in 98% purity. The characterization of the other two components failed due to lack of isolated material. 4-Allyl-2,7-dimethylocta-2,3,5,6-tetraene (**14**): ^{1}H NMR ($CDCl_3$) δ 1.70 (s, 6H, terminal allene CH_3), 1.72 (d, ^5J = 2.9 Hz, 6H, term. allene CH_3), 2.73 (m, 2H, -CH_2-), 4.98 (m, 1H, term. allyl H), 5.12 (m, 1H, term. allyl H), 5.56 (sept, ^5J = 2.9 Hz, 1H, int. allene H), 5.77–5.88 ppm (m, 1H, int. allyl H). ^{13}C NMR ($CDCl_3$) δ 20.8 (q, 4C, allene CH_3), 35.3 (t, 1C, -CH_2-), 91.6 (d, 1C, int. allene C(H), 96.7 (s, 1C, int. allene C), 98.6 and 98.7 (s, 2C, ext. allene C), 114.9 (t, 1C, =CH_2), 136.9 (d, 1C, =CH-), 202.4 and 202.8 ppm (s, 2C, central allene C). 7-Allyl-4,4,9-trimethyldeca-1,7,8-trien-5-yne (**20**): IR (film): $\tilde{\upsilon}$ = 2977 (vs), 2933 (s), 2874 (m), 2200 (w), 1890 (w), 1727 (s), 1384 (s), 1364 (s), 1185 (s), 1139 (s), 1090 (s), 1069 (s), 1020 (s), 997 (s), 919 cm^{-1} (s). UV (acetonitrile) λ_{max} (lg ε): 192 (4.02), 222 nm (3.70). ^{1}H NMR ($CDCl_3$) δ

1.18 (s, 6H, gem-dimethyl), 1.70 (s, 6H, allene CH_3), 2.18 (d, ^3J = 6.7 Hz, 2H, -CH_2-), 2.79 (d, ^3J = 6.7 Hz, 2H, -CH_2-), 5.01–5.10 (m, 4H, =CH_2), 5.78–5.87 and 5.87–5.99 ppm (m, 2H, =CH-). ^{13}C NMR ($CDCl_3$) δ 20.4 (q, 2C, gem-dimethyl), 29.2 (q, 2C, allene CH_3), 31.9 (s, 1C, quart. C), 40.1 (t, 1C, -CH_2-), 48.1 (t, 1C, -CH_2-), 77.7 (s, 1C, -C≡C-), 87.5 (s, 1C, -C≡C-), 97.3 and 97.6 (s, 2C, allene C), 116.1 and 117.4 (t, 2C, =CH_2), 135.8 and 136.0 (d, 2C, =CH-), 206.9 ppm (s, 1C, central allene C). HRMS: calcd 214.1721; found, 214.1721.

d) Formylation of **2**: preparation of **22**: A solution of **2** (670 mg, 5 mmol) was lithiated as described in GP-1. Subsequently the generated carbanion was quenched with dimethyl-formamide (36 mg, 5 mmol) and the reaction mixture worked up by treatment with 1 N hydrochloric acid (10 mL). An oily raw product was obtained after solvent removal (600 mg, 75%), which was nearly pure (>90 % by GC analysis). Kugelrohr distillation (bp 50 °C, 0.1 mbar) under loss of material (isolated: 380 mg, 48 %) provided analytically pure (99% GC analysis) 2,7-dimethyl-4-formylocta-2,3,5,6-tetraene (**22**) as a yellow oil. IR (film): ῦ = 2983 (s), 2910 (s), 2854 (s), 1949 (w), 1715 (s), 1682 (vs), 1555 (s), 1364 (s), 1170 (m), 1067 cm^{-1} (m). UV (acetonitrile) λ_{max} (lg ε): 192 (4.16), 228 (3.93, sh), 304 nm (3.65). ^{1}H NMR ($CDCl_3$) δ 1.71 (d, ^5J = 2.8 Hz, 6H, allene CH_3), 1.97 (s, 6H, allene CH_3), 5.59 (sept, ^5J = 2.8 Hz, 1H, allene H), 9.49 (s, 1H, -CHO). ^{13}C NMR ($CDCl_3$) δ 19.5 and 20.0 (q, 4C, allenic CH_3), 81.0 (d, 1C, int. allenic C(H), 97.8, 102.9, 106.3 (s, 3C, allenic quart. C), 190.9 (d, 1C, -*C*HO), 204.6 and 216.0 ppm (s, 2C, central allenic C). MS (70 eV) *m*/*z* (%): 162 (8) $[M]^+$, 147 (8), 119 (10), 91 (11), 86 (62), 84 (100), 47 (17), 40 (13).

e) Reaction of **4** with acetone: preparation of **23**, **24**, and **25**: To a solution of **2** (2.68 g, 20 mmol) in anhydrous THF (50 mL) TMEDA (2.77 g, 24 mmol) was added at −35 °C under nitrogen followed by a solution of *n*-butyllithium in hexane (19.2 mL, 1.25 mol, 25 mmol). After stirring for 2 h at this temperature, acetone (dried over $CaCl_2$ and molecular sieves 4 Å) was added dropwise and the reaction was warmed to room temp. overnight. Water was added

(20 mL) followed by dichloromethane (100 mL). The organic phase was thoroughly washed with water, dried ($MgSO_4$), and the solvent was removed in vacuo. Addition of pentane to the remaining yellow oil resulted in the precipitation of a colorless solid, which was shown to possess structure **25** (see below). Removal of the pentane gave a yellow oil from which the substrate **2** could be recovered (150 mg). The remaining product mixture (2.00 g, 49 % raw yield) contained four components: starting material **2** (25%, GC analysis), bisallene **23** (13%), allenyne **24** (9%), and diyne **25** (39%, increasing order of elution). This mixture was subjected to kugelrohr distillation (up to 100 °C, 0.1 mbar), which provided enriched fractions of the different reaction products, which could be purified by preparative gas chromatography (Carbowax, 3 m, 150–200 °C). In the end 325 mg (10%) of **23**, 180 mg (4%) of **24**, and 410 mg (8%) of **25** resulted, which could be analyzed by spectroscopy. **23**: IR (film): $\tilde{\upsilon}$ = 3548 (br. OH, m), 2981 (vs), 2935 (s), 2909 (s), 1963 (w), 1447 (s), 1377 (s), 1363 (s), 1340 (s), 1168 (m), 953 cm^{-1} (m). UV (acetonitrile) λ_{max} (lg ε): 192 (4.25, sh), 210 (4.29), 232 nm (4.08, sh). ^1H NMR ($CDCl_3$) δ 1.35 (s, 6H, gem-dimethyl), 1.70 (s, 6H, allenic CH_3), 1.73 (d, ^5J = 3.2 Hz, 6H, allenic CH_3), 2.30 (s, 1H, OH; showed H/D-exchange), 5.52 ppm (sept., ^5J = 3.2 Hz, 1H, allenic H). ^{13}C NMR ($CDCl_3$) δ 20.4 (q, 2C, allenic CH_3), 20.5 (q, 2C, allenic CH_3), 29.2 (q, 2C, gem-dimethyl), 71.5 (s, 1C, quart. C), 88.1 (d, 1C, allenic C(H), 97.9 (s, 1C, ext. allenic C), 99.1 (s, 1C, ext. allenic C), 108.0 (s, 1C, int. allenic C), 199.6 (s, 1C, central allenic C), 201.9 ppm (s, 1C, central allenic C). MS (70 eV) *m*/*z* (%): 192 (11) $[M]^+$, 177 (84), 159 (22), 149 (58), 135 (30), 133 (32), 121 (32), 119 (78), 105 (48), 91 (100), 79 (32), 43 (56), 41 (48). HRMS: calcd 192.1514; found, 192.1514. **24**: IR (film): $\tilde{\upsilon}$ = 3420 (br. OH, s), 2982 (s), 2935 (s), 2810 (m), 1762 (m), 1378 (s), 1230 (s), 1164 (s), 1168 (m), 913 (s), 733 (vs), 647 cm^{-1} (s). UV (acetonitrile) λ_{max} (lg ε): 206 (3.74), 232 nm (3.71, sh). ^1H NMR ($CDCl_3$) δ 1.27 and 1.28 (s, 12H, gem-dimethyl), 1.36 (s, 6H, gem-dimethyl). 1.55 (s, 2H, OH, shows H/D-exchange), 1.75 ppm (s, 6H, allenic CH_3). ^{13}C NMR ($CDCl_3$) δ 20.7 (q, 2C, gem-dimethyl), 24.9 and 25.3 (4C, gem-dimethyl), 29.3 (q, 2C, allenic CH_3), 41.5 (s, 1C,

C($(CH_3)_2$), 72.0 and 75.0 (s, 2C, -C≡C-), 97.0 and 98.0 (s, 2C, ext. and int. allenic C), 210.0 ppm (s, 1C, central allenic C). MS (70 eV) *m*/*z* (%): 250 (1) $[M]^+$, 209 (14), 207 (12), 193 (20), 191 (30), 177 (31), 174 (33), 159 (50), 149 (32), 119 (46), 107 (40), 105 (46), 91 (48), 59 (80), 43 (100), 41 (44). HRMS: calcd 250.1932; found, 250.1932. **25**: IR (film): ʋ̃ = 3370 (vs, br. OH, s), 2980 (vs), 2940 (m), 1370 (m), 1140 (s), 960 cm^{-1} (w). UV (acetonitrile) λ_{max} (lg ε): 192 nm (4.38, sh). ^1H NMR ($CDCl_3$) δ 1.22 (s, 12H, gem-dimethyl), 1.24 (s, 12H, gem-dimethyl). 1.84 ppm (s, 2H, OH, shows H/D-exchange). ^{13}C NMR ($CDCl_3$) δ 24.3 (q, 4C, gem-dimethyl), 25.1 (4C, gem-dimethyl), 41.4 (s, 2C, *C*($(CH_3)_2$), 66.7 (s, 2C, *C*($(CH_3)_2$), 74.2 and 83.8 ppm (s, 4C, C≡C-). MS (70 eV) *m*/*z* (%): 217 (2) $[M^+ - 33]$, 177 (15), 159 (25), 134 (12), 119 (21), 91 (22), 59 (100), 43 (33), 41 (16). $C_{16}H_{26}O_2 \cdot H_2O$ (268.40): calcd C 71.60, H 10.52; found, 71.29, H 10.41.

f) Oxidation of **2**, **7**, **5, 11,** and **12** with MMPP:

General procedure 2 (GP-2): To a solution of the bisallene (usually 1 mmol) in methanol (20 mL) was added MMPP and the mixture was stirred for 5 d at room temp. The free acid was filtered off and pentane (50 mL) was added to the filtrate. The organic layer was washed thoroughly with water and the solvent was removed by rotary evaporation at room temp. The raw product was purified by kugelrohr distillation (30 °C, 0.1 mbar).

5,5-Dimethyl-4-(epoxy-1-methylethenyliden)-2-cyclopenten-1-one (**37**): From **2** (134 mg, 1 mmol) and MMPP (2.1 g, 85%, 5 mmol) according to GP-2: 110 mg (66%) of **37** was obtained as a colorless oil. IR (film): ʋ̃ = 2976 (s), 2935 (s), 2877 (m), 1722 (vs), 1692 (s), 1380 (s), 1185 (s), 1118 (s), 1051 (s), 905 (m), 894 cm^{-1} (s). - UV (acetonitrile) λ_{max} (lg ε): 224 nm (3.76). ^1H NMR ($CDCl_3$) δ 1.02 and 1.07 (s, 6H, gem-dimethyl), 1.44 and 1.47 (s, 6H, gem-dimethyl), 6.35 (d, ^3J = 6.0 Hz, 1H, -CH=), 7.19 ppm (d, ^3J = 6.0 Hz, 1H, -CH=). ^{13}C NMR ($CDCl_3$) δ 20.4 and 21.8 (q, 2C, gem-dimethyl), 22.8 and 22.9 (q, 2C, gem-

dimethyl), 44.9 (s, 1C, CH_3), 64.8 (s, 1C, CH_3), 75.5 (s, 1C, *C*(O,C), 135.3 (d, 1C, -CH=), 158.4 (d, 1C; -CH=), 212.1 ppm (s, 1C, C=O). MS (70 eV) *m/z* (%): 166 (14) $[M]^+$, 156 (20), 124 (20), 93 (18), 80 (92), 79 (54), 73 (100), 57 (24), 56 (22). HRMS: calcd 166.0993; found, 166.0993. The spectroscopic data of **36**, produced in 80% yield when 1 equivalent of MMPP was used for the oxidation, agree with those observed in the original epoxidation experiment [5].

2,3,5,5-Tetramethyl-4-(methylethenyliden)-2-cyclopenten-1-one (**38**): From **7** (162 mg, 1 mmol) and MMPP (291 mg, 85%, 0.5 mmol) according to GP-2 after 3 d at room temp: mixture (GC analysis) of substrate **7** (24%), **38** (68%), and **39** (see below) 8%. The substrate could be removed in vacuo (0.3 mbar, 30 °C, 2 h), leaving the mixture of **38** and **39**. The structure of the former was established by subtraction of the NMR signals of **39** (see below) from the NMR spectra of the **38**/**39** mixture. ^{1}H NMR ($CDCl_3$) δ 1.21 (s, 6H, gem-dimethyl), 1.79, 1.98, 2.0, 2.29 ppm (s, 12H, 4 x CH_3); ^{13}C NMR ($CDCl_3$) δ 8.9 (q, 1C, =C-*C*H_3), 15.4 (q, 1C, =C-*C*H_3), 22.9 (q, 2C, gem-dimethyl), 23.3 and 24.3 (q, 2C, =C(*C*H_3)$_2$), 46.8 (s, 1C, *C*(CH_3)$_2$), 131.0 and 135.3 (s, 2C, *C*=*C*), 141.8 (s, 1C, O=C-*C*=C), 162.6 (s, 1C, O=C-*C*=*C*), 211.3 ppm (s, 1C, *C*=O); MS (70 eV) *m/z* (%): 178 (62) $[M]^+$, 163 (24), 150 (16), 135 (100), 119 (19), 93 (12), 91 (20), 81 (16), 79 (14), 41 (19).

2,3,5,5-Tetramethyl-4-(epoxy-1-methylethenyliden)-2-cyclopenten-1-one (**39**): From **7** (670 mg (5 mmol) and MMPP (870 mg, 15 mmol, 85%) according to GP-2: After 2 h the oxidation to **39** was complete (GC analysis); after kugelrohr distillation (0.1 mbar, 45 °C): 120 mg (62%) of pure **39**, slightly yellow oil. IR (film): ῦ = 2790 (s), 2932 (s), 1706 (vs), 1467 (m), 1386 (s), 1070 (m), 733 cm^{-1} (s). - UV (acetonitrile) λ_{max} (lg ε): 195 (3.58), 243 nm (4.03). ^{1}H NMR ($CDCl_3$) δ 1.00 and 1.18 (s, 6H, gem-dimethyl), 1.47 and 1.55 (s, 6H, gem-dimethyl), 1.76 and 1.94 ppm (s, 6H, -C(CH_3)=C(CH_3). ^{13}C NMR ($CDCl_3$) δ 9.0 (q, 1C, -C(*C*H_3)=), 15.4 (q, 1C, -C(*C*H_3)=), 21.4, 22.4, 32.2, 26.0 (q, 4C, gem-dimethyl), 45.4 (s,

1C, gem-$C(CH_3)_2$, 65.2 (s, 1C, gem-$C(CH_3)_2$), 76.7 (s, 1C, *C*(O,C), 140.2 and 163.8 (s, 2C, -C=C-), 210.7 ppm (s, 1C, C=O). MS (70 eV) *m*/*z* (%): 194 (6) $[M]^+$, 152 (58), 137 (14), 109 (16), 108 (100), 107 (25), 93 (64), 86 (34), 84 (56), 77 (12), 43 (20), 41 (19). HRMS: calcd 194.1306; found, 194.13064.

3,5,5-Trimethyl(epoxy-1-methylethenyliden)-2-cyclopenten-1-one (**40**) and 2,5,5-Trimethyl-(epoxy-1-methylethenyliden)-2-cyclopenten-1-one (**41**): From **5** (740 mg, 5 mmol) and 1.45 g (25 mmol, 85%) of MMPP according to GP-2: 590 mg (65%), slightly yellow oil. The mixture could not be separated by preparative gas chromatography, ratio **40**:**41** = 5:1 by ^{1}H NMR analysis. The structure assignments are based on the product mixture. Spectral data of the mixture: IR (film): ʋ̃ = 2970 (s), 2933 (s), 1714 (vs), 1466 (m), 1381 (m), 1117 (m), 914 cm^{-1} (m). UV (acetonitrile) λ_{max} (lg ε): 196 (3.71), 234 nm (3.74). **40**: ^{1}H NMR ($CDCl_3$) δ 1.06 and 1.23 (s, 6H, gem-dimethyl), 1.52 and 1.60 (s, 6H, gem-dimethyl), 2.09 (d, ^4J = 1.4 Hz, 3H, $=CCH_3$), 6.18 ppm (q, ^4J = 1.4 Hz, 1 H, =CH). ^{13}C NMR ($CDCl_3$) δ 18.3 (q, 1C, $C(CH_3)=$), 21.0, 22.7, 23.0, 25.5 (q, 4C, gem-dimethyl), 46.9 (s, 1C, gem-$C(CH_3)_2$), 65.5 (s, 1C, *C*(O,C), 75.6 (s, 1C, *C*(O,C), 134.5 (d, 1C, -C=), 173.0 (s, 1C, -CH=), 210.7 ppm (s, 1C, C=O). GC–MS (70 eV) *m*/*z* (%): 180 (8) $[M]^+$, 138 (35), 122 (17), 107 (32), 94 (45), 79 (100), 43 (23), 41 (19). HRMS: calcd 180.1150; found, 180.1150. **41**: ^{1}H NMR ($CDCl_3$) δ 1.08 and 1.19 (s, 6H, gem-dimethyl), 1.47 and 1.50 (s, 6H, gem-dimethyl), 1.87 (d, ^4J = 1.2 Hz, 3H, $=CCH_3$), 6.86 ppm (q, ^4J = 1.2 Hz, 1H, =CH). ^{13}C NMR ($CDCl_3$) δ 10.6 (q, 1C, $C(CH_3)=$), 20.7, 21.9, 23.0, 23.2 (q, 4C, gem-dimethyl), 45.1 (s, 1C, gem-$C(CH_3)_2$), 64.4 (s, 1C, *C*(O,C), 74.6 (s, 1C, *C*(O,C), 143.9 (s, 1C, -C=), 151.9 (d, 1C, -CH=), 212.1 ppm (s, 1C, C=O). GC–MS (70 eV) *m*/*z* (%): 180 (4) $[M]^+$, 138 (13), 122 (39), 107 (12), 94 (100), 79 (45), 43 (16).

5,5-Dimethyl-2,3-trimethylsilyl-4-(1-methylethenyliden)-2-cyclopenten-1-one (**45**): From **11** (139 mg, 0.5 mmol) and MMPP (147 mg, 0.25 mmol) according to GP-2. After 2 d at room temp., a mixture was obtained, which contained substrate **11** (20%), mono-oxidation product

45 (54%), and bis-oxidation product **46** (24%, GC-analysis). Whereas the separation of **11** from this mixture was successful (kugelrohr distillation), the separation of **45** and **46** (see below) failed. Still, the decisive analytical data could be extracted from the spectra. **45**: ^{1}H NMR ($CDCl_3$) δ 0.26 (s, 9H, $Si(CH_3)_3$), 0.34 (s, 9H, $Si(CH_3)_3$), 1.18 (s, 6H, gem-dimethyl), 1.91 and 1.93 ppm (s, 6H, $=C(CH_3)_2$). ^{13}C NMR ($CDCl_3$) δ 0.5 (q, 3C, $Si(CH_3)_3$), 1.8 (q, 3C, $Si(CH_3)_3$), 22.1 and 26.5 (q, 2C, $=C(\mathit{C}H_3)_2$), 23.8 (q, 2C, gem-dimethyl), 48.0 (s, 1C, gem-$\mathit{C}(CH_3)_2$), 129.3 (s, 1C, C=CTMS), 149.9 (s, 1C, $=\mathit{C}(CH_3)_2$), 156.0 (s, 1C, $\mathit{C}=C(CH_3)_2$), 185.5 (s, 1C, C=CTMS), 210.9 ppm (s, 1C, C=O).

5,5-Dimethyl-2,3-trimethylsilyl-4-(epoxy-1-methylethenyliden)-2-cyclopenten-1-one (**46**): The epoxidation (according to GP-2) was repeated with **11** (670 mg, 5 mmol) and MMPP (2.0 g, 3.4 mmol, 85%). After 10 d at room temp., the starting material had been consumed completely. Work-up (see GP-2, distillation at 60 °C, 0.1 mbar) provided 530 mg (71%) of **46** as yellow oil. IR (film): ῦ = 2962 (s), 2928 (s), 1695 (vs), 1465 (m), 1375 (m), 1250 (s), 1091 (s), 844 (vs), 687 cm^{-1} (s). UV (acetonitrile) λ_{max} (lg ε): 192 (4.01), 318 nm (2.83). ^{1}H NMR ($CDCl_3$) δ 0.27 (s, 9H, $(CH_3)_3Si$), 0.29 (s, 9H, $Si(CH_3)_3$), 1.03 and 1.12 (s, 6H, gem-dimethyl), 1.44 and 1.49 ppm (s, 6H, gem-dimethyl). ^{13}C NMR ($CDCl_3$) δ 0.8 (q, 3C, $Si(CH_3)_3$), 2.9 (q, 3C, $Si(CH_3)_3$), 22.4, 22.9, 24.6, 25.9 (q, 4C, gem-dimethyl), 46.9 (s, 1C, gem-$\mathit{C}(CH_3)_2$), 65.3 (s, 1C, *C*(O,C), 84.2 (s, 1C, *C*(O,C), 160.8 (s, 1C, -C=), 188.5 (d, 1C, -CH=), 213.2 ppm (s, 1C, C=O). MS (70 eV) *m*/*z* (%): 310 (8) $[M]^+$, 295 (10), 238 (10), 225 (20), 224 (100), 205 (22), 73 (26). HRMS: calcd 310.1784; found, 310.17864.

3-(*tert*-Butyldimethylsilyl)-4-(1-methylethenyliden)-5,5-dimethyl-2-cyclopeneten-1-one (**48**): According to GP-2 bisallene **12** (362 mg, 10 mmol) of **12** was epoxidized with MMPP (580 mg, 10 mmol, 85%) for 3 d. GC-analysis of the product mixture showed that 47 % of the substrate had not reacted. The ^{1}H NMR spectrum of the raw product mixture displayed only signals between δ = −1 and 2 ppm. The ^{13}C NMR spectrum showed the following signals

agreeing with structure proposal **47**: ^{13}C NMR ($CDCl_3$) δ −1.5 (s, 4C, $Si(CH_3)_2$), 18.7 and 20.8 (s, 2C, *C*$(CH_3)_3$), 29.1 and 30.0 (q, 6C, C(*C*$H_3)_3$), 48.5 (s, 1C, *C*$(CH_3)_2$), 129.1 (s, 1C, = *C*$(CH_3)_2$), 151.9 (s, 1C, C=*C*Si), 157.7 (s, 1C, *C*=$C(CH_3)_2$), 187.6 (s, 1C, C=*C*Si), 215.3 ppm (s, 1C, C=O). Preparative gas chromatography (Carbowax, 3 m, 240 °C) yielded 30 mg (11%) of a slightly yellow oil to which structure **48** was assigned due to the following spectroscopic and analytical data: IR (film): ῦ = 2959 (s), 2932 (s), 2860 (s), 1696 (vs), 1486 (m), 1255 (m), 1076 (m), 910 (s), 732 (vs), 671 cm^{-1} (m). UV (acetonitrile) λ_{max} (lg ε): 208 (3.94), 308 nm (3.93). ^{1}H NMR ($CDCl_3$) δ 0.36 (s, 6H, $(CH_3)_2Si$), 0.93 (s, 9H, $Si(CH_3)_3$), 1.24 (s, 6H, gem-dimethyl), 1.96 and 1.98 (s, 6H, =*C*$(CH_3)_2$), 6.45 ppm (s, 1H, =CH-). ^{13}C NMR ($CDCl_3$) δ −1.2 (q, 2C, Si(*C*$H_3)_2$), 18.0 (s, 1C, Si*C*$(CH_3)_3$), 23.0 (q, 1 C, gem-dimethyl), 23.3 (q, 1C, =C(*C*$H_3)_2$), 27.5 (q, 3C, SiC(*C*$H_3)_3$), 48.1 (s, 1C, gem-*C*$(CH_3)_2$), 134.1 (s, 1C, =*C*$(CH_3)_2$), 141.6 (d, 1C, -CH=), 147.0 (s, 1C, *C*=C), 171.6 (s, 1C, C=*C*Si), 213.0 ppm (s, 1C, C=O). MS (70 eV) *m/z* (%): 264 (12) $[M]^+$, 207 (100), 179 (28), 137 (23), 123 (17), 99 (28), 73 (76), 59 (24). HRMS: calcd 264.1909; found, 264.1909.

g) Oxidation of **3** with air: A solution of **3** (670 mg, 5 mmol) in dichloromethane (20 mL) was shaken under an atmosphere of oxygen for 4 d. GC–MS-analysis showed that one compound had been formed in 20% yield; 35% of the substrate did not react. Further oxidation did not increase the yield of the oxidation product. Isolation of the latter by preparative GC (SE-30, 3 m, 100 °C) yielded analytically pure 5-*tert*-butyl-4-(1-methylethenyliden)-2-cyclopenten-1-one (**44**; 50 mg, 7%), yellow oil. IR (film): ῦ = 2961 (s), 2908 (m), 2871 (m), 1702 (vs), 1556 (m), 1283 (m), 1163 (m), 906 cm^{-1} (m). UV (acetonitrile) λ_{max} (lg ε): 215 (3.72), 270 (3.89), 292 nm (3.39, sh). ^{1}H NMR ($CDCl_3$) δ 1.00 (s, 9H, $(CH_3)_3$), 2.50 (m, 1H, -CH-), 5.26 and 5.41 (m, 2H, $=CH_2$), 6.18 and 7.66 ppm (m, 2H, -CH=CH-). ^{13}C NMR ($CDCl_3$) δ 27.9 (q, 3C, C(*C*$H_3)_3$), 28.0 (s, 1C, *C*$(CH_3)_3$), 57.7 (d, 1C, CH), 114.6 (t, 1C, $=CH_2$), 134.7 (d, 1C, CH=CH), 147.9 (s, 1C, *C*$=CH_2$), 159.5 (d, 1C, CH=CH), 208.6 ppm (s, 1C, C=O). MS

(70 eV) *m/z* (%): 150 (6) $[M]^+$, 135 (16), 108 (12), 107 (26), 94 (100), 91 (24), 79 (18), 57 (48), 41 (26). HRMS: calcd 150.1045; found, 150.1045.

h) Addition of HCl and HBr to **2** and **5**:

Hydrochlorination of **2**: To a solution of **2** (1.34 g, 10 mmol) in anhydrous ether (50 mL) was slowly added a solution of gaseous HCl in ether (4.2 mL of a 2.33 m solution) under nitrogen at −70 °C. After completion of the addition the reaction mixture was stirred at −30 °C for 5 h, and then brought to room temp. overnight. The solution was washed with brine, dried over $MgSO_4$, and the solvent was removed by rotary evaporation. The remaining brown oil (1.2 g) was separated/purified by preparative GC (20% Apiezonon Chromosorb W, 150 °C), leading to a yellow oil (64%, total yield: 45%) and a colorless side product (20%). The former consists of **55** and **56** (R = H, *trans*/*cis*-ratio 10:1; mp ca. 35 °C) by cap. gas chromatography, the latter is *m*-cymene (**57**, R = H) as shown by comparison with an authentic sample (Aldrich). The separation of **55** and **56** (R = H, X = Cl) by preparative GC failed. Spectral data of the **55**/**56**-mixture: IR (CCl_4): $\tilde{\upsilon}$ = 2980 (s), 2920 (s), 2850 (m), 1450 (m), 1360 (m), 1150 (s), 970 cm^{-1} (m). UV (acetonitrile) λ_{max} (lg ε): 190 (3.73), 276 (1.38, sh), 283 (4.49), 294 nm (4.38). ^1H NMR ($CDCl_3$) δ 1.78 (s, 6H, $(CH_3)_2C$=CH, **56**), 1.83 (s, 6H, $(CH_3)_2C$=CH, **55**), 1.91 (s, 3H, $(CH_3)_2C$=CCl, **55**), 1.94 (s, 3H, $(CH_3)_2C$=CCl, **55**), 1.97 (s, 3H, $(CH_3)_2C$=CCl, **56**), 2.02 (s, 3H, $(CH_3)_2C$=CCl, **56**), 5.95 (d, ^3J = 11.1 Hz, 1H, 6-H, **55**), 6.45 (d, ^3J = 14.4 Hz, 1H, 4-H, **55**), 6.77 (dd, ^3J = 14.2, ^3J = 11.2 Hz, 1H, 5-H, **55**). The corresponding olefinic signals of **56** were not intense enough to allow a reliable assignment. ^{13}C NMR ($CDCl_3$) δ 18.4, 18.6, 20.6, 21.2, 23.0, 26.2, 26.3, 26.5 (q, CH_3 of **55**/**56**), 123.5, 124.9, 125.0, 126.9, 127.6, 130.2 (d, =CH- of **55**/**56**), 119.7, 126.5, 130.9, 133.8, 137.3, 137.5 ppm (s, =CR- of **55**/**56**). MS (70 eV) *m/z* (%): 172 (22) $[M]^+$, 170 (66) $[M]^+$, 157 (16), 155 (70), 91 (100), 77 (71), 65 (28), 53 (29).

Hydrobromination of **2**: To a solution of **2** (1.34 g, 10 mmol) in diethyl ether (50 mL) was slowly added aqueous hydrobromic acid (1.27 mL of 47% aq. HBr, 10 mmol) at 0 °C under nitrogen. After 3 h at that temp., the reaction was worked up as above to provide a brown oil as the raw product (1.8 g); separation by preparative GC (see above) provided a mixture of **55** and **56** (R = H, X = Br, 53%, ratio **55**/**56**: 20:1, mp 43 °C) and as a second fraction **57** (27%). The further separation of **55** and **56** failed. Spectroscopic data of the **55**/**56**-mixture (R = H, X = Br): IR (CCl_4): $\tilde{\upsilon}$ = 2970 (m), 2920 (m), 1440 (m), 1375 (m), 1150 (w), 945 (m), 910 cm^{-1} (s). UV (acetonitrile) λ_{max} (lg ε): 203 (3.64), 263 (4.28), 274 (4.48, sh), 283 (4.59), 295 nm (4.50). ^{1}H NMR ($CDCl_3$) δ 1.78 (s, 6H, $(CH_3)_2C$=CH, **56**), 1.82 (s, 6H, $(CH_3)_2C$=CH, **55**), 1.91 (s, 3H, $(CH_3)_2C$=CBr, **55**), 1.94 (s, 3H, $(CH_3)_2C$=CBr, **56**), 1.97 (s, 3H, $(CH_3)_2C$=CBr, **55**), 2.02 (s, 3H, $(CH_3)_2C$=CBr, **56**), 5.95 (d, ^3J = 10.7 Hz, 1H, 6-H, **55**),6.45 (d, ^3J = 14.2 Hz, 1H, 4-H, **55**), 6.77 (dd, ^3J = 14.3, ^3J = 11.0 Hz, 1H, 5-H, **55**). The corresponding olefinic signals of **56** (R = H, X = Br) were not intense enough to allow a reliable assignment. ^{13}C NMR ($CDCl_3$) δ 18.6, 18.7, 20.6, 21.2, 22.0, 23.0, 26.2, 26.5 (q, CH_3 of **55**/**56**), 123.5, 124.9, 124.8, 126.8, 127.4, 130.2 (d, =CH- of **55**/**56**), 119.5, 126.5, 130.8, 133.8, 137.5, 137.5 ppm (s, =CR- of **55**/**56**). MS (70 eV) *m/z* (%): 216 (56) $[M]^+$, 214 (66) $[M]^+$, 201 (8), 199 (8), 135 (93), 119 (87), 105 (83), 91 (100), 77 (67), 65 (26), 53 (28). HRMS: calcd 214.036, found, 214.036.

Hydrochlorination of **5**: To a solution of **5** (740 mg, 5 mmol) in anhydrous ether (100 mL) was added at −45 °C under nitrogen hydrochloric acid (2.27 mL, 5 mmol, 2 m solution) in ether (25 mL). After stirring for 5 h at this temp., the mixture was warmed to room temp. overnight and worked up as above. The raw mixture (0.8 g, 80%) was separated by preparative GC (80% Apiezon on Chromosorb W) and provided **55** (R = CH_3, X = Cl) in 28% (250 mg) and **56** (R = CH_3, X = Cl) in 3% yield (30 mg), both diastereomers being more than 99% pure (cap. GC analysis). (*E*)-3-Chloro-2,5,7-trimethylocta-2,4,6-triene (**55**): IR (film):

ῡ = 2971 (s), 2931 (vs), 2913 (vs), 2855 (s), 1444 (s), 1376 (s), 1153 (m), 888 cm^{-1} (s). UV (acetonitrile) λ_{max} (lg ε): 192 (4.03), 256 nm (4.05). ^{1}H NMR ($CDCl_3$) δ 1.72 (s, 6H, $(CH_3)_2C$=CCl), 1.79 (d, ^{4}J = 1.3 Hz, 3H, C=C$(CH_3)_2$), 1.82 (d, ^{4}J = 1.3 Hz, 3H, C=C$(CH_3)_2$), 1.90 (m, 3H, =C(CH_3)CH=), 5.65 and 5.73 ppm (m, 2H, -HC=C). ^{13}C NMR ($CDCl_3$) δ 18.9, 19.8, 21.3, 21.7, 26.9 (q, 5C, CH_3), 123.5, 130.1, 135.0, 138.2 (s, 4C, quart. C=), 125.3, 127.7 ppm (d, 2C, =CH-). MS (70 eV) *m*/*z* (%): 186 (13) $[M]^+$, 184 (40) $[M]^+$, 171 (31), 169 (92), 150 (10), 149 (98), 134 (80), 133 (96), 121 (34), 119 (78), 107 (70), 105 (90), 91 (100), 77 (58), 65 (32), 53 (32). HRMS: calcd 184.1018; found, 184.1018. (*Z*)-3-Chloro-2,5,7-trimethylocta-2,4,6-triene (**56**): IR (film): ῡ = 2970 (s), 2930 (s), 2913 (s), 2857 (m), 1692 (m), 1442 (s), 1377 (m), 1131 (s), 1112 (s), 870 cm^{-1} (m). UV (acetonitrile) λ_{max} (lg ε): 192 (4.02), 214 (3.80, sh), 256 nm (3.85). ^{1}H NMR ($CDCl_3$) δ 1.64 and 1.68 (s, 6H, $(CH_3)_2C$=CCl), 1.76 and 1.86 (s, 6H, C=C$(CH_3)_2$), 1.91 (s, 3H, =C(CH_3)CH=), 5.67 and 5.71 ppm (m, 2H, -HC=C). ^{13}C NMR ($CDCl_3$) δ 19.9, 21.6, 21.7, 24.0, 27.0 (q, 5C, CH_3), 123.6, 130.0, 135.2, 138.1 (s, 4C, quart. C=), 124.4 and 124.5 ppm (d, 2C, =CH-). MS (70 eV) *m*/*z* (%): 186 (12) $[M]^+$, 184 (42) $[M]^+$, 170 (22), 169 (99), 149 (88), 133 (100), 119 (65), 106 (64), 105 (76), 91 (80), 77 (44), 65 (22), 20 (32).

Addition of bromine to **2**: To a solution of **2** (670 mg, 5 mmol) in trichloromethane (50 mL) was added under vigorous stirring bromine (1.6 g, 10 mmol) in trichloromethane (25 mL) at −40 °C. After stirring for a further 30 min at this temperature the reaction mixture was brought to room temperature and neutral aluminum oxide (50 g) and water (20 mL) were added. The suspension was stirred for 2 h, the aluminum oxide was removed by filtration, the phases were separated, and the organic phase was dried ($MgSO_4$). The solvent was removed in vacuo, leaving the reaction product (1.5 g) as highly viscous oil. When this was taken up in trichloromethane, the adduct **60** precipitated as an amorphous solid, which was recrystallized from trichloromethane: 0.52 g (32%) of **60**, colorless cubes, mp 123–125 °C. IR (KBr): ῡ =

3280 (br., s), 3220 (vs), 2980 (m), 1460 (m), 1410 (m), 1375 (m), 1260 (m), 1170 (s), 925 (s), 855 cm^{-1} (m). UV (acetonitrile) λ_{max} (lg ε): 251 (4.40, sh), 257 (4.46), 264 nm (4.35, sh). ^{1}H NMR ($CDCl_3$) δ 1.54 (s, 12H, CH_3), 1.55 (s, 2H, OH, exch. with D_2O), 6.99 ppm (s, 2H, =CH). ^{13}C NMR ($CDCl_3$) δ 29.1 (q, 4C, CH_3), 74.6 (s, 2C, *C*(CH_3)$_2$), 123.7 (d, 2C, =CH), 141.2 ppm (s, 2C, -CBr=). MS (70 eV) *m*/*z* (%): 330 (16) $[M]^+$, 328 (34) $[M]^+$, 326 (17) $[M]^+$, 313 (26), 295 (28), 231(84), 229 (80), 214 (20), 167 (32), 150 (52), 135 (40), 107 (45), 91 (55), 59 (100). $C_{10}H_{16}Br_2O_2$ (328.04): calcd 36.61, H 4.92; found, C 36.66, H 4.75.

j) Addition of iodine to **2** and **3**: To a solution of **2** (134 mg, 1 mmol) in carbon tetrachloride (10 mL) iodine (254 mg, 1 mmol) was added in small portions at −20 °C. After a few minutes (the iodine color did not disappear completely) the solvent was removed by rotary evaporation and the remaining oil taken up in petrol ether. In the cold (freezer) the iodine addition product **63** precipitates as a colorless solid: 320 mg (82%), mp 143 °C (decomp.). IR (KBr): $\tilde{\upsilon}$ = 3420 (m), 2995 (s), 1250 (m), 1150 (s), 890 (m), 850 cm^{-1} (m). UV: the product was insoluble in the common UV solvents. ^{1}H NMR ($CDCl_3$) δ 2.13 and 2.14 (s, 12H, CH_3), 6.49 ppm (s, 2H, HC=CH); ^{13}C NMR ($CDCl_3$) δ 21.6 and 33.0 (q, 4C, CH_3), 99.0 (s, 2C, =CI), 135.2 (d, 2C, =CH), 142.6 ppm (s, 2C, =*C*(CH_3)$_2$). MS (70 eV) *m*/*z* (%): 388 (1) $[M]^+$, 335 (2), 254 (100), 127 (46), 96 (20), 43 (26). $C_{10}H_{14}I_2$ (388.09): calcd 30.95, H 3.64; found, C 30.68, H 3.50.

In an analogous experiment **3** (134 mg, 1 mmol) was reacted with iodine (200 mg, 0.79 mmol) at −20 °C. GC–MS analysis of the raw product mixture showed that two iodine adducts had been formed in 60 and 4% yield, respectively, the rest being starting material **3**. After the solvent had been removed by rotary evaporation and the remainder been taken up in petrol ether, an amorphous colorless product precipitated at −15 °C from this solution, to which we assign structure **64** (mixture of isomers). This solid product (mp 60 °C, decomp.) contained at least two diastereomeric diiodides. Separation of these compounds by preparative gas chromatography and/or column chromatography failed. The following analytic data hence

refer to the mixture of adducts. IR (KBr): ῡ = 3450 (m), 2962 (vs), 1690 (s), 1593 (m), 1477 (s), 1462 (m), 1366 (s), 1337 (s), 1238 (m), 1068 (s), 1008 (s), 993 (s), 893 (m), 850 cm^{-1} (m). UV (acetonitrile; qual.) λ_{max} (lg ε): 196, 246, 283, 325 nm (sh). ^{1}H NMR ($CDCl_3$) δ 1.24 (s, 9H, $(CH_3)_3$), 6.00 and 6.47 (d, ^3J = 13.5 Hz, 2H, -CH=CH-), 6.04 (s, 1H, *t*-BuHC=), 6.44 and 6.59 ppm (ps-s, 2H, =CH_2). ^{13}C NMR ($CDCl_3$) δ 31.3 (q, 3C, $(CH_3)_3$), 37.8 (s, 1C, *C*$(CH_3)_3$), 96.1 and 106.1 (s, 2C, =CI), 130.7 (t, 1C, =CH_2), 137.0 and 140.2 (d, 2C, -CH=CH-), 157.0 ppm (d, 1C, *t*-Bu*C*=). MS (70 eV) *m*/*z* (%): 388 (3) $[M]^+$, 261 (11), 134 (60), 119 (100), 92 (14), 91 (26), 77 (17), 57 (78), 41 (51). $C_{10}H_{14}I_2$(388.09): calcd 30.95, H 3.64; found, C 30.99, H 3.61.

k) Addition of chlorosulfonyl isocyanate (CSI, **66**) to **2**: A solution of CSI (6.09 g, 43 mmol) in tetrachloromethane (10 mL) was added to a solution of **2** (5.72 g, 43 mmol) in tetrachloromethane (10 mL) at 0 °C under stirring. The reaction mixture was stirred at this temp. until the isocyanate absorption at 2260 cm^{-1} in the IR spectrum had vanished (ca. 4 h). The mixture was poured onto ice, the organic phase was separated, and washed thoroughly with small portions of water. The aqueous phases were combined and cooled (5 °C), and colorless crystals (210 mg, 2.3%) precipitated from the solution; to these, structure **75** was assigned (see below). The solvent of the organic phase was removed in vacuo, and the chlorosulfonyl group of the remaining raw product (7.4 g) was removed by hydrolysis (treatment of the crude product with a mixture of diethyl ether and aqueous sodium sulfite solution (25%) to which an aqueous potassium hydroxide solution (10%) had been added; pH = 8). After stirring for 15 min, the organic phase was separated and dried over $MgSO_4$. Solvent removal by rotary evaporation provided an oily residue, which was taken up in acetone. On standing in the cold, colorless crystals (1.18 g, 32%) precipitated from this solution, to which we assign structure **72**. Exposed to air, the compound rapidly polymerizes. Spectroscopic data of the free β-lactam **72**: IR (KBr): ῡ = 3430 (s), 3259 (s), 2987 (m), 1945

(w), 1728 (s), 1685 (s), 1620 (m), 1443 (s), 1402 (s), 1396 (m), 1298 (m), 1183 (vs), 1158 (s), 1095 (s), 1030 (s), 820 (m), 625 (s), 576 (s), 501 cm^{-1} (s). UV (ethanol) λ_{max} (lg ε): 205 (3.45), 294 nm (3.76). ^{1}H NMR ($CDCl_3$) δ 1.79 (d, ^5J = 2.7 Hz, 6H, =C(CH_3)$_2$), 1.86 (s, 6H, gem-dimethyl), 5.66 (dsept, ^5J = 2.7, ^3J = 11.7 Hz, 1H, allenic H), 6.79 ppm (d, ^3J = 11.7 Hz, 1H, -CH=). ^{13}C NMR ($CDCl_3$) δ 19.6 and 24.1 (q, 4C, CH_3), 73.9 (s, 1C, *C*(CH_3)$_2$), 86.8 (d, 1C, int. allenic C), 98.8 (s, 1C, =*C*(CH_3)$_2$), 131.4 (d, 1C, =*C*H), 138.2 (s, 1C, =C), 157.0 (s, 1C, centr. allenic C), 212.6 ppm (s, C=O). MS (70 eV) *m*/*z* (%): 177(12) $[M]^+$, 176 (70), 163 (12), 162 (100), 149 (35), 148 (20), 110 (86), 92 (38), 82 (45), 68 (56), 67 (46), 42 (34), 41 (33).

Spectroscopic data of the trienamide **75**: IR (KBr): ῦ = 3615 (s), 3603 (s), 3515 (vs), 3307 (m), 1679 (vs), 1655 (s), 1648 (s), 1629 (s), 1447 (vs), 1262 (vs), 1206 (s), 1043 (vs), 947 (m), 607 (m), 566 cm^{-1} (m). UV (methanol) λ_{max} (lg ε): 206 (3.57), 275 (4.15), 284 (4.27), 296 nm (4.17). ^{1}H NMR (CD_3OD) δ 1.90, 1.93, 1.97 (s, 12H, CH_3), 6.66, 6.79 ppm (d, ^3J = 15.2 Hz, 2H, -CH=CH-). ^{13}C NMR (CD_3OD) δ 18.1, 19.3, 21.4, 21.8 (q, 4C, CH_3), 122.8 and 123.6 (d, 2C, -CH=CH-), 125.3 (s, 1C, =CCl-), 131.2, 132.9, 135.0 (s, 3C, remaining olefinic C), 170.4 ppm (s, 1C, C=O). MS (70 eV) *m*/*z* (%): 215 (6) $[M]^+$, 213 (17) $[M]^+$, 178 (100), 177 (26), 162 (19), 160 (14), 145 (14), 144 (19), 135 (18), 133 (22), 129 (32), 117 (16), 105 (18), 91 (26), 19 (14), 77 (18), 64 (20), 41 (18).

References

[1]] Skattebøl,L.; Solomon, S. *J. Am. Chem. Soc*. 1965, *87*, 4506-4513.

[2] Skattebøl, L. *Tetrahedron Lett*. **1961**, 167-172.

[3] Ruitenberg, K.; Kleijn,H.; Meijer, J.; Westrmijze, H.; Vermeer, P. *Recl. Trav. Chim. Pays-Bas*, **1982**, *101*, 405-409.

[4] Jacobs, T. L.; Prempree, P. *J. Am. Chem. Soc*. **1967**, *89*, 6179-6182.

[5] Pasto, D. J.; Yang, S.-H.; Muellerleile, J. A. *J. Org. Chem*. **1992**, *57*, 2976-2978.
